# Integration of Chemical Probes and Degradomics Reveals That Cathepsin B Functions Primarily as a Dipeptidyl Carboxypeptidase in Macrophages

**DOI:** 10.1002/cmdc.70530

**Published:** 2026-10-29

**Authors:** Kai‐Qi Yek, Francesca L. Apolloni, Bangyan Xu, Nghi H. Nguyen, Alexander R. Ziegler, Nichollas E. Scott, Brad E. Sleebs, Laura E. Edgington‐Mitchell

**Affiliations:** ^1^ Department of Biochemistry & Pharmacology, Bio21 Molecular Science and Biotechnology Institute The University of Melbourne Parkville VIC Australia; ^2^ Molecular Imaging and Nano Therapeutics Laboratory Baker Heart and Diabetes Institute Melbourne VIC Australia; ^3^ Centre for Cardiometabolic mRNA Therapy Baker Heart and Diabetes Institute Melbourne VIC Australia; ^4^ Department of Cardiometabolic Health University of Melbourne Parkville VIC Australia; ^5^ The Walter and Eliza Hall Institute of Medical Research Parkville VIC Australia; ^6^ Department of Medical Biology The University of Melbourne Parkville VIC Australia; ^7^ Department of Microbiology & Immunology Peter Doherty Institute The University of Melbourne Parkville VIC Australia

**Keywords:** activity‐based probes, C‐terminomics, cathepsin B, dipeptidyl carboxypeptidase, proteases

## Abstract

Cathepsin B (Ctsb) is a lysosomal cysteine protease implicated in proteostasis and disease, but its endogenous substrate specificity and activity in intact cells remain incompletely defined. Here, we applied an integrated chemical biology and degradomics approach to profile Ctsb function in RAW264.7 macrophages using the irreversible inhibitor Z‐RK‐AOMK. Z‐RK‐AOMK treatment effectively inhibited intracellular Ctsb activity while also perturbing the broader cysteine cathepsin network, including altered activities of Ctsx and Ctsl, and accumulation of an active higher molecular weight Ctss species consistent with altered Ctss maturation. Despite these changes in cysteine cathepsin activity, Z‐RK‐AOMK treatment produced limited changes in global protein abundance and N‐terminal processing. In contrast, C‐terminomic profiling revealed enrichment of cleavage events occurring two and four residues upstream of native protein C‐termini. This pattern supports a predominant role for Ctsb as a dipeptidyl carboxypeptidase in cells and highlights C‐terminome profiling as a sensitive functional readout of its activity. Finally, we developed a fluorescent activity‐based probe, BODIPY‐RK‐AOMK, which selectively labels active Ctsb in cell lysates and live cells, enabling visualization of its predominantly lysosomal localization. Together, these findings define the cellular processing signature of Ctsb in macrophages and establish a complementary activity‐based probe for mapping its activity in biological systems.

## Graphical abstract

An integrated chemical biology and degradomics approach reveals the proteolytic landscape of cathepsin B in mouse macrophages. Z‐RK‐AOMK perturbs the cysteine cathepsin network and reveals that cathepsin B predominantly exhibits dipeptidyl carboxypeptidase activity in cells. An activity‐based probe BODIPY‐RK‐AOMK was developed for visualizing active cathepsin B in cells.

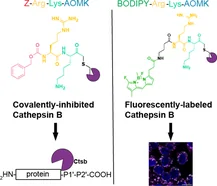

## 1 Introduction

Cathepsin B (Ctsb) is a lysosomal cysteine protease that is ubiquitously expressed across human tissues, highlighting its fundamental role in cellular homeostasis [1]. Under physiological conditions, Ctsb contributes to intracellular and extracellular protein turnover, antigen processing, hormone activation, and bone remodeling [2]. Beyond its canonical lysosomal functions, Ctsb has also been detected in cytosolic [3], extracellular [4], and nuclear [5, 6, 7] compartments under conditions of cellular stress or disease. In these extralysosomal locations, Ctsb has been implicated in programmed cell death, inflammation, tumor progression, and neurodegenerative disorders [8, 9]. Notably, Ctsb remains catalytically active at both acidic and neutral pH [10, 12], indicating it can maintain proteolytic function within nonlysosomal environments. These observations suggest that Ctsb fulfills diverse context‐dependent roles beyond bulk lysosomal protein degradation.

Unlike most cathepsins, which primarily function as endopeptidases [13], Ctsb possesses both endopeptidase and exopeptidase activities [10]. The latter is mediated by an occluding loop within its protein structure that enables sequential removal of dipeptides from protein C‐termini [10, 14]. Biochemical studies using purified enzymes and synthetic peptide libraries have shown that Ctsb preferentially functions as a dipeptidyl carboxypeptidase under both acidic and neutral pH conditions [10, 11].

Despite detailed in vitro characterization of its substrate specificity [10], how Ctsb contributes to endogenous protein processing in intact cells remains poorly understood. Previous studies have identified a limited number of candidate substrates, including extracellular matrix proteins (e.g., type IV collagen, laminin, and fibronectin [15]) and the cell adhesion protein E‐cadherin [16]. These investigations were largely targeted and based on recombinant proteins, with biased proteome‐wide analyses remaining scarce. A notable exception is the degradomics study by Prudova et al. [17], which applied terminal amine isotopic labeling of substrates (TAILS) [18] to pancreatic tumors from wild‐type and Ctsb‐deficient mice, identifying pyruvate kinase M2, endoplasmic reticulum chaperone BiP, and prothymosinalpha as putative endogenous substrates. However, as TAILS‐based analysis exclusively captures N‐terminal proteolytic events, it cannot detect proteolytically generated C‐termini, particularly those generated near the native protein terminus. Consequently, C‐terminal cleavage events generated by the dipeptidyl carboxypeptidase activity of Ctsb have not been identified, leaving a major aspect of its endogenous proteolytic function unexplored.

Addressing this gap requires chemical tools capable of selectively perturbing Ctsb activity in living systems while allowing unbiased profiling of downstream proteolytic events. Among available inhibitors, CA‐074 has been widely used to inhibit Ctsb activity but is limited by poor cellular permeability, while its cell‐permeable counterpart CA‐074Me has been reported to also inhibit other cathepsins, including cathepsin L [19, 20, 21, 22]. To overcome these limitations and enable interrogation of compartment‐specific Ctsb activity, Z‐RK‐AOMK was developed by Yoon et ‍al. [11] to selectively inhibit human CTSB under neutral pH conditions (Figure 1A). The inhibitor contains an acyloxymethylketone (AOMK) electrophile that covalently modifies the catalytic cysteine residue, an Arg‐Lys (R‐K) dipeptide, and an N‐terminal benzyloxycarbonyl (Z) protecting group [11, 12] (Figure 1A). In vitro, Z‐RK‐AOMK exhibits selectivity for CTSB, with an IC_50_ of 20 nM at pH 7.2 compared with 1500 nM at pH 4.6 [11]. At acidic pH, 16 µM Z‐RK‐AOMK inhibits other cathepsins, albeit to a lesser degree than CTSB [11]. At neutral pH, inhibition of CTSS (IC_50_ = 2200 nM), CTSV (IC_50_ = 440 nM), and CTSC (IC_50_ = 850 nM) was observed, although Z‐RK‐AOMK is at least 22‐fold more potent against CTSB (IC_50_ = 20 nM) [11]. However, the selectivity of Z‐RK‐AOMK toward endogenous cathepsin pools in living cells remains to be defined [12]_._

**FIGURE 1:**
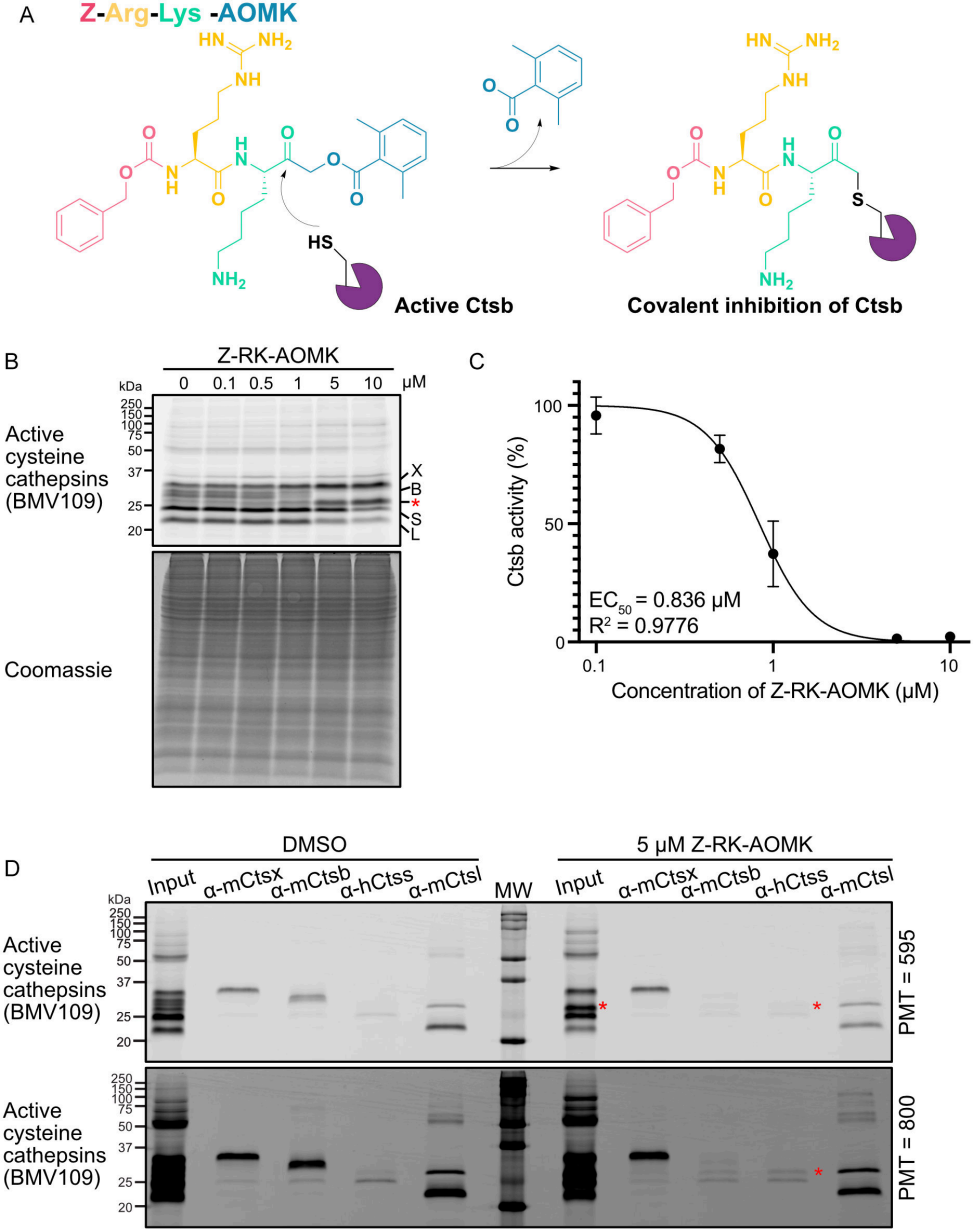
Z‐RK‐AOMK inhibits Ctsb in RAW264.7 cells. (A) Schematic of irreversible inhibition of Ctsb by Z‐Arg‐Lys‐AOMK (Z‐RK‐AOMK), in which the catalytic cysteine residue reacts with the AOMK electrophile. (B) RAW264.7 cells were treated with increasing concentrations of Z‐RK‐AOMK (0–10 µM) for 16 h. Residual cysteine cathepsin activity was then detected by labeling live cells with 1 µM BMV109 for 4 h. Cells were harvested, lysed in PBS containing 0.1% Triton X‐100, and analyzed by SDS‐PAGE followed by in‐gel Cy5 fluorescence imaging. The gel was then stained with Coomassie Blue as a loading control. Corresponding raw images are provided in the Supporting Information. (C) Ctsb activity was quantified by densitometry analysis of the BMV109‐labeled Ctsb band (as shown in B) and normalized to that of DMSO control (100%). Data from three biological replicates were fitted in Prism 10 using a four‐parameter variable slope model with bottom and top constraints of 0% and 100%, respectively. Data represent mean ± SD. The resulting fit showed *R* ^2^ = 0.9776, yielding an EC_50_ of 0.836 µM for intracellular Ctsb inhibition by Z‐RK‐AOMK in RAW264.7 cells. (D) Selectivity of Z‐RK‐AOMK was assessed by immunoprecipitation of Ctsx, Ctsb, Ctss, and Ctsl from BMV109‐labeled cells treated with DMSO or 5 µM Z‐RK‐AOMK. Immunoprecipitants were resolved by SDS‐PAGE and analyzed by in‐gel Cy5 fluorescence to confirm the identity of BMV109‐labeled bands. The top panel is shown at optimal exposure, while the bottom panel is overexposed to reveal fainter bands. Corresponding raw images are provided in the Supporting Information.

Here, we apply Z‐RK‐AOMK as both a chemical perturbation tool and a scaffold for derivatization into an activity‐based probe to visualize active Ctsb in situ. The inhibition of intracellular Ctsb by Z‐RK‐AOMK and its effects on other cysteine cathepsins were assessed using cathepsin activity‐based probes. Using quantitative proteomics together with no‐enrichment identification of cleavage events (NICE) [23, 24], an unbiased degradomics approach that captures endogenous protein termini [25], we define the proteomic and proteolytic consequences of Ctsb inhibition in RAW264.7 macrophages. Finally, we synthesize a fluorescent derivative of the Z‐RK‐AOMK scaffold, BODIPY‐RK‐AOMK, to enable direct visualization of active Ctsb in intact cells.

## 2 Results and Discussion

### 2.1 Validation of Z‐RK‐AOMK Inhibition of Cathepsin B in RAW264.7 Cells

Z‐RK‐AOMK has previously been evaluated in human neuroblastoma cells, inhibiting intracellular CTSB activity with an EC_50_ of approximately 50 nM, showing no detectable cytotoxicity at concentrations up to 20 µM [12]. As macrophages express abundant Ctsb and other cysteine cathepsins, we sought to investigate the selectivity of Z‐RK‐AOMK in the mouse macrophage cell line RAW264.7 [26].

RAW264.7 cells were treated overnight with increasing concentrations of Z‐RK‐AOMK and subsequently labeled with the pan‐cysteine cathepsin activity‐based probe BMV109 [27] (Figure ‍1B). BMV109 covalently labels active cysteine cathepsins and becomes fluorescent upon binding, enabling visualization of protease activity. Analysis of probe‐labeled lysates revealed a concentration‐dependent reduction in labeling of the protein corresponding to the size of the active Ctsb single chain, migrating between the 25 and 37 kDa molecular weight markers (Figure 1B). Complete loss of Ctsb labeling was observed following treatment with 5 or 10 µM Z‐RK‐AOMK, indicating near‐complete inhibition of intracellular Ctsb activity (Figure 1B).

Quantification of Ctsb labeling yielded an apparent cellular EC_50_ of 0.836 µM (Figure 1C and Table S1). Although this value is higher than that previously reported in human neuroblastoma cells (50 nM) [12], complete inhibition of intracellular Ctsb activity was achieved at 5 µM in RAW264.7 cells. This difference may reflect cell‐specific factors, including variation in Ctsb expression levels, compound uptake or accumulation, or differences in assay readouts. Species‐dependent differences between human and mouse Ctsb may also contribute to the observed discrepancy.

### 2.2 Z‐RK‐AOMK Alters Activity of Other Cysteine Cathepsins

Following Z‐RK‐AOMK treatment, we also observed changes in the intensity of additional BMV109‐labeled bands (Figure 1B). To confirm the identity of bands detected by the BMV109 probe, individual cysteine cathepsins were immuno precipitated from BMV109‐labeled cell lysates using specific antibodies (Figure 1D). Immunoprecipitation of Ctsb confirmed that the corresponding BMV109‐labeled band was abolished following treatment with 5 µM Z‐RK‐AOMK (Figure 1D), reaffirming complete inhibition of intracellular Ctsb activity. Ctsl pulldown revealed reduction in the activity of its heavy chain (~23 kDa), but not the single chain (~26 kDa), while Ctsx activity conversely increased after treatment. Additionally, immunoprecipitation of Ctss revealed an additional higher molecular weight (>25 kDa) Ctss species (Figure 1D, marked with asterisk) that co‐migrated with the single‐chain form of Ctsl. As the single‐chain Ctsl band showed no visible change following Z‐RK‐AOMK treatment, the increased intensity of the band marked with an asterisk was primarily attributed to Ctss (Figure 1D).

We next utilized immunoblots to determine whether changes in cathepsin activity were associated with altered abundance. Ctsx (also known as Ctsz) activity was significantly increased in the Z‐RK‐AOMK treated cells (Figure 2A,G first panel; 3.077‐fold‐change, *p* value <0.0001, and Table S2), without a corresponding change in its total abundance (Figure 2B,H first panel and Table S2). This may reflect functional adaptation within the lysosomal protease network, which is predicted due to the reciprocal compensation of Ctsb and Ctsx [28, 29, 30]. Given the overlapping carboxypeptidase activities of these enzymes, with Ctsx acting predominantly as a mono‐ but also dipeptidyl carboxypeptidase [31], and Ctsb functioning mainly as a dipeptidyl carboxypeptidase as well as an endopeptidase [11], redistribution of substrate processing capacity may contribute to these changes.

**FIGURE 2:**
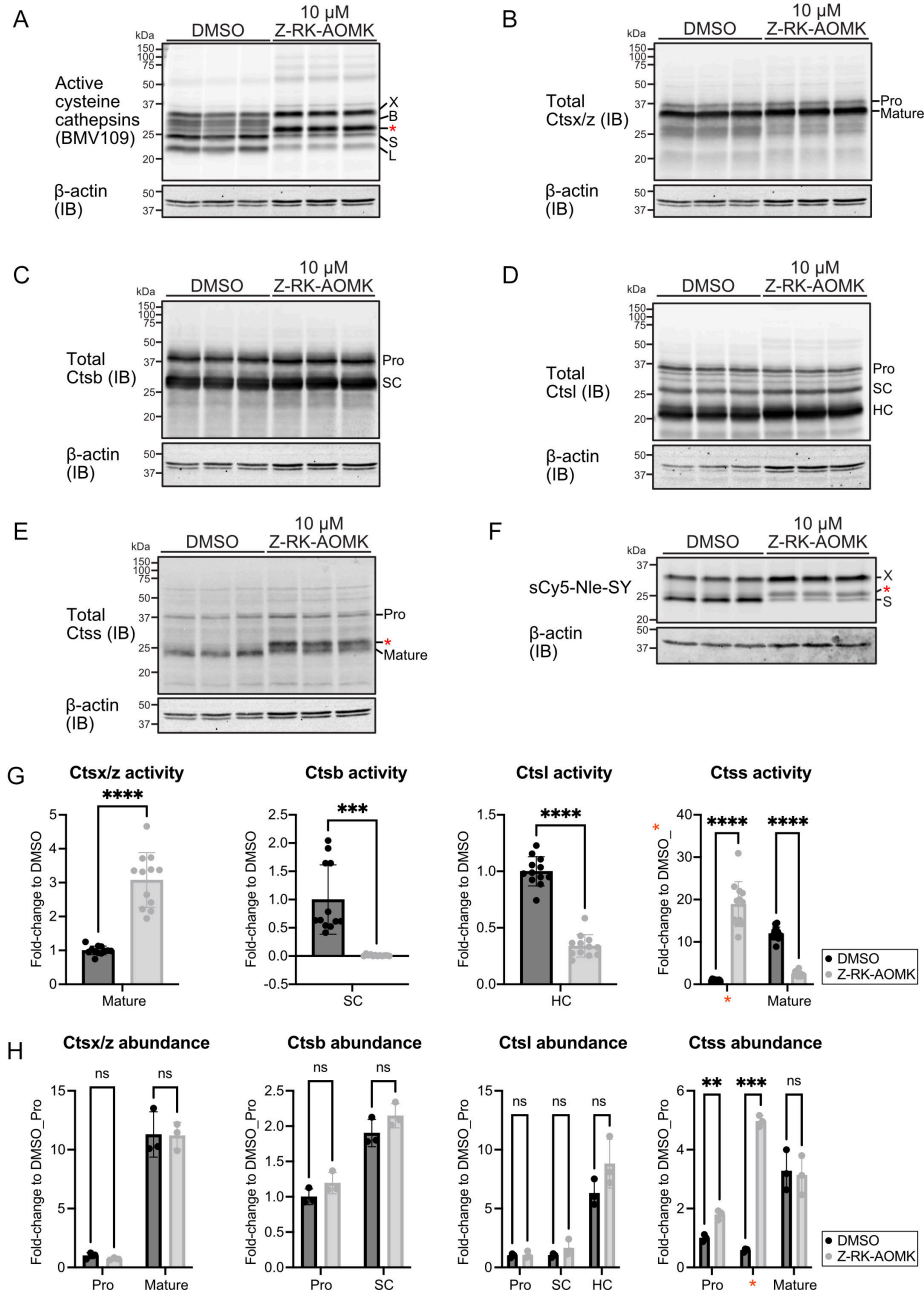
Activities and abundance of cysteine cathepsins upon Z‐RK‐AOMK treatment. Corresponding raw images are provided in the Supporting Information. (A) RAW264.7 cells were treated with DMSO or 10 µM Z‐RK‐AOMK for 16 h. Residual cysteine cathepsin activity was then detected by labeling live cells with 1 µM BMV109 for 4 h. Cells were harvested, lysed in PBS containing 0.1% Triton X‐100, and analyzed by SDS–PAGE followed by in‐gel Cy5 fluorescence imaging and immunoblotting for β‐actin. (B–E) The same lysates were analyzed by immunoblotting for Ctsx (B), Ctsb (C), Ctsl (D), and Ctss (E), with β‐actin included as a loading control in each blot. (F) RAW264.7 cells were treated with DMSO or 10 µM Z‐RK‐AOMK for 16 h. Residual active Ctsx and Ctss were then labeled in live cells with 1 µM sCy5‐Nle‐SY for 4 h. Cells were harvested, lysed in PBS containing 0.1% Triton X‐100, and analyzed by SDS‐PAGE followed by in‐gel Cy5 fluorescence imaging. (G) Quantification of cathepsin activity by densitometry analysis of the BMV109‐labeled bands shown in (A). Signal intensities were normalized to Ponceau S total protein stain and shown as fold‐change relative to DMSO‐treated cells (set to 1). Data represent mean ± SD from *n* = 12 biological replicates. Statistical significance was assessed using unpaired t‐tests in Prism 10. ***p < 0.001, ****p < 0.0001. (H) Quantification of cathepsin abundance from the immunoblots shown in (B–E). Signal intensities for each cathepsin form were normalized to Ponceau S total protein stain and shown as fold‐change relative to the corresponding pro‐form in DMSO‐treated cells (set to 1). Data represent mean ± SD from *n* = 3 biological replicates. Statistical significance was assessed using unpaired t‐tests in Prism 10. NS = not significant, **p < 0.01, ***p < 0.001.

Inhibition of Ctsb by Z‐RK‐AOMK (Figure 2A,G second panel; 0.0115‐fold‐change, *p‐*value = 0.0002, and Table S2) was not associated with changes in its total abundance (Figure 2C,H second panel and Table S2). The heavy‐chain form of Ctsl showed significantly reduced activity upon Z‐RK‐AOMK treatment (Figure ‍2A,G third panel; 0.34‐fold‐change, *p‐*value < 0.000001, and Table S2) without changes in protein abundance (Figure 2D,H third panel and Table S2).

### 2.3 Z‐RK‐AOMK Alters Ctss Maturation and Generates a Glycosylated Higher Molecular Weight Species

Immunoprecipitation with anti‐Ctss antibody revealed enrichment of the >25 kDa species (Figure 1D, marked with an asterisk) in Z‐RK‐AOMK‐treated lysates. Immunoblot analysis further demonstrated accumulation of this higher molecular weight Ctss species following Z‐RK‐AOMK treatment (Figure 2E,H last panel with asterisk; 4.384‐fold increase relative to DMSO, *p‐*value = 0.00021, and Table S2). In parallel, abundance of the Ctss proform also increased upon Z‐RK‐AOMK treatment (Figure 2E,H last panel; 1.793‐fold‐change to DMSO proform, *p*‐value = 0.003204, and Table S2). Together, these data suggest that Ctss maturation is perturbed following Z‐RK‐AOMK treatment, leading to accumulation of alternative Ctss species.

Given that immunoprecipitation did not reveal substantial changes in the abundance of the single‐chain Ctsl species (Figure ‍1D), we attributed theBMV109‐labeled band marked with an asterisk primarily to Ctss (Figure 2A). Quantification of BMV109 labeling showed markedly increased signal from this higher molecular weight Ctss species following Z‐RK‐AOMK treatment (Figure 2G last panel; 18.9‐fold‐change relative to DMSO, *p‐*value <0.000001, and Table S2), consistent with its increased abundance (Figure 2H last panel and Table S2). In contrast, BMV109 labeling of the canonical mature Ctss species was significantly reduced following Z‐RK‐AOMK treatment (Figure 2D last panel; 9.515‐fold difference relative to DMSO, *p‐*value < 0.000001, and Table S2), despite no detectable change in its abundance (Figure ‍2H last panel and Table S2).

We verified these observations with the sulfoxonium ylide activity‐based probe sCy5‐Nle‐SY [32] that selectively targets Ctss and Ctsx. As with BMV109, labeling of Ctsx was markedly increased upon Z‐RK‐AOMK treatment (Figures 2F, S1A; 1.969 fold‐change, *p‐*value = 0.0499, and Table S2), concomitant with the appearance of the higher molecular weight Ctss species (Figures 2F, S1B; 0.213‐fold increase, *p‐*value = 0.029494, and Table S2), whereas labeling of the canonical Ctss was significantly reduced (Figures 2F, S1B; 0.06141 fold‐change to DMSO, *p‐*value = 0.008062, and Table S2).

To further characterize the higher molecular weight Ctss species, we next attempted to affinity purify it using the biotinylated activity‐based probe DCG‐04 [33]. Streptavidin blotting of DCG‐04‐labeled RAW264.7 lysates confirmed labeling of Ctsx, Ctsb, and Ctss, with complete loss of Ctsb labeling following 10 µM Z‐RK‐AOMK treatment and concurrent appearance of the non‐canonical Ctss species (Figure 3A). Proteomic analysis of streptavidin‐enriched samples confirmed Ctsb, Ctss, and Ctsx as the DCG‐04labeled cathepsins (Figure 3B, and Table S3), with no Ctsb peptides detected in the Z‐RK‐AOMK‐treated condition (Figure 3C and Table S3). Only a single Ctss peptide mapping to the central region of the catalytic domain was detected in both DMSO and Z‐RK‐AOMK‐treated samples (Figure 3D and Table S3), which did not reveal any information to distinguish the canonical and higher molecular weight Ctss species.

**FIGURE 3:**
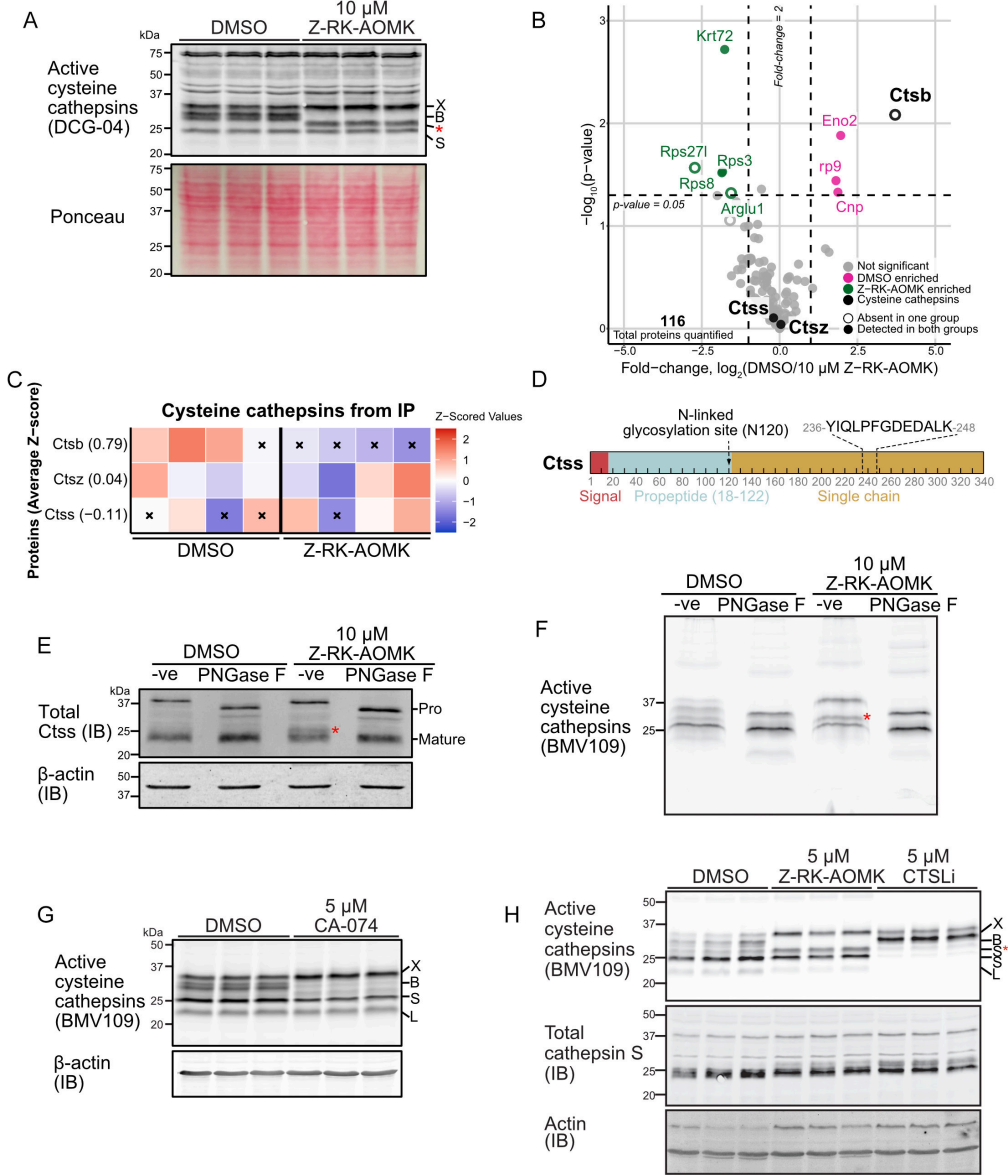
Affinity enrichment of active cathepsins labeled by DCG‐04. (A) RAW264.7 cells were treated with DMSO or 10 µM Z‐RK‐AOMK for 16 h. Residual active cysteine cathepsins were then labeled in live cells with 1 µM DCG‐04 for 4 h. Cells were harvested, lysed in PBS containing 0.1% Triton X‐100, and analyzed by SDS–PAGE followed by immunoblotting with streptavidin‐800. Corresponding raw images are provided in the Supporting Information. (B) RAW264.7 cells were treated with DMSO or 10 µM Z‐RK‐AOMK for 16 h and then labeled with 1 µM DCG‐04 for 4 h to label residual active cysteine cathepsins in live cells. Cells were harvested and lysed in PBS containing 0.1% Triton X‐100. DCG‐04–labeled proteins were enriched using streptavidin agarose beads and analyzed by mass spectrometry‐based proteomics. Log_2_ fold‐change in protein abundance is plotted against −Log_10_ *p*‐value on the scatter plot (*n* = 4 biological replicates). (C) Heatmap showing cathepsins identified by proteomic analysis of DCG‐04 immunoprecipitants. Protein abundance is displayed as *Z*‐scores calculated from the label‐free quantification (LFQ) intensities. Replicates with imputed values are indicated by a cross within the corresponding box. (D) Schematic representation of the mouse Ctss protein sequence showing the signal peptide, propeptide, and mature single‐chain region. The predicted N‐linked glycosylation site and the peptide identified by proteomics in (B) are indicated. (E) RAW264.7 cells were treated with DMSO or 10 µM Z‐RK‐AOMK for 16 h. Cells were harvested, lysed in PBS containing 0.1% Triton X‐100, and treated with PNGase F before analysis by SDS‐PAGE and immunoblotting for Ctss and β‐actin. Corresponding raw images are provided in the Supporting Information. (F) RAW264.7 cells were treated with DMSO or 10 µM Z‐RK‐AOMK for 16 h and live‐labeled with BMV109. Cells were harvested, lysed in PBS containing 0.1% Triton X‐100, and treated with PNGase F before analysis by SDS–PAGE and scanning for Cy5 fluorescence. Corresponding raw images are provided in the Supporting Information. (G) RAW264.7 cells were treated with DMSO or 5 µM CA‐074 for 16 h. Residual active cysteine cathepsins were then labeled in live cells with 1 µM BMV109 for 4 h. Cells were harvested, lysed in PBS containing 0.1% Triton X‐100, and analyzed by SDS–PAGE followed by in‐gel Cy5 fluorescence imaging and immunoblotting for β‐actin. Corresponding raw images are provided in the Supporting Information. (H) RAW264.7 cells were treated with DMSO, 5 µM CA‐074 or 5 µM CTSLi for 16 h. Residual active cysteine cathepsins were then labeled in live cells with 1 µM BMV109 for 4 h. Cells were harvested, lysed in PBS containing 0.1% Triton X‐100, and analyzed by SDS–PAGE followed by in‐gel Cy5 fluorescence imaging and immunoblotting for Ctss and β‐actin. Corresponding raw images are provided in the Supporting Information.

The higher molecular weight Ctss species could result from differential processing during maturation within the endolysosomal system following Z‐RK‐AOMK treatment or from altered post‐translational modification, such as differential glycosylation. Ctss contains a single predicted N‐linked glycosylation site at N120 [23], located two residues upstream of the annotated end of the propeptide region (Figure 3D). Therefore, the canonical mature Ctss species is predicted to lack this glycosylation site following removal of the propeptide. To investigate whether the higher molecular weight Ctss species was glycosylated, DMSO and Z‐‍RK‐AOMK‐treated cell lysates were incubated with PNGase F, an enzyme that removes N‐linked glycans from glycoproteins [34]. As expected, the canonical mature Ctss species showed no detectable molecular weight shift following PNGase F treatment, consistent with the absence of N‐linked glycosylation. In contrast, the Ctssproform showed reduced molecular weight following PNGase F treatment, confirming the presence of N‐linked glycans (Figure 3E). The higher molecular weight Ctss species that accumulated following Z‐RK‐AOMK treatment was no longer observed after PNGase F treatment, likely collapsing to the size of the mature species (Figure 3E), indicating that this species contains an N‐linked glycan. This PNGase‐induced Ctss shift was also observed with BMV109 labeling (Figure 3F).

These findings suggest that the higher molecular weight Ctss species contains an N‐linked glycan, most likely at N120, consistent with incomplete or altered propeptide processing during Ctss maturation. The de‐glycosylated species was indistinguishable from the mature form, suggesting the intermediate form differs by only a few residues plus the N‐glycan. The Ctss propeptide is normally removed through autocatalytic processing during maturation [35, 36], and inhibition of Ctss activity could therefore disrupt this process. Consistent with this, similar higher molecular weight CTSS species have been reported following treatment of human promyeloid HL‐60 cells with the broad‐spectrum cysteine protease inhibitor E64d [37], inhibition of recombinant CTSS with the selective inhibitor LHVS [38], or inhibition of recombinant CTSS by the endogenous cysteine cathepsin inhibitor cystatin C [39], supporting the idea that inhibition of CTSS activity perturbs its normal maturation. This higher molecular weight Ctss species was not detected in RAW264.7 cells using BMV109 labeling following inhibition of Ctss with LHVS for 1 h, which inhibited Ctss and Ctsl [40], despite its accumulation after E64d treatment in HL‐60 cells [37]. Furthermore, inhibition of Ctsb alone with CA‐074 in RAW264.7 cells did not result in the accumulation of this Ctss species (Figure 3G) [41]. By contrast, overnight treatment with Cathepsin L inhibitor I (Z‐FF‐FMK), which inhibited Ctsl and both Ctss species at 5 µM in RAW264.7 cells, but not Ctsb, was able to induce accumulation of the Ctss intermediate to an even greater extent than Z‐RK‐AOMK (Figure 3H). Together, these observations suggest that Ctsl, but not Ctsb, may contribute to this intermediate processing step during Ctss maturation in RAW264.7 cells.

### 2.4 Proteomic Consequences of Sustained Ctsb Inhibition

To investigate the cellular consequences of Ctsb inhibition, we performed proteomic analyses following treatment of RAW264.7 cells with Z‐RK‐AOMK. Although Z‐RK‐AOMK affects the activity of several cysteine cathepsins, intracellular Ctsb activity was fully inhibited following treatment with 5 µM Z‐‍RK‐AOMK, whereas other cathepsins were only partially affected. Therefore, these analyses provide an opportunity to examine the cellular consequences of Ctsb inhibition while recognizing that contributions from other affected cathepsins cannot be completely excluded.

Employing the No‐enrichment Identification of Cleavage Events (NICE) workflow [23], we examined changes in protein abundance and endogenous proteolytic processing in RAW264.7 cells treated with 5 µM Z‐RK‐AOMK for 24, 48, and 72 h. As NICE is an enrichment‐free approach, the resulting dataset simultaneously provides information on both protein abundance and endogenous proteolytic processing. Briefly, cell lysates were subjected to dimethyl labeling of primary amines, including protein N‐termini and lysine side chains, prior to trypsin digestion and mass spectrometry analysis [23]. Since trypsin‐generated N‐termini are formed after dimethyl labeling, they remain unlabeled, allowing native and neo‐ (endogenous protease‐generated) N‐termini to be distinguished from internal trypsin peptides based on the presence of the dimethyl tag.

For protein abundance analysis, the raw data were searched using fully enzymatic (tryptic) search parameters to maximize confidence in protein quantification [42]. Despite quantification of more than 6200 proteins, relatively few proteins were differentially regulated following Z‐RK‐AOMK treatment across all timepoints (Figures 4A–C and S2A and Table S4). Notably, the majority of significantly altered proteins were increased in abundance in Z‐RK‐AOMK‐treated cells (Figure 4A–C and Table S4). This suggests that inhibition of Ctsb activity by Z‐RK‐AOMK potentially inhibits protein degradation, consistent with its role in protein turnover [2, 17].

**FIGURE 4:**
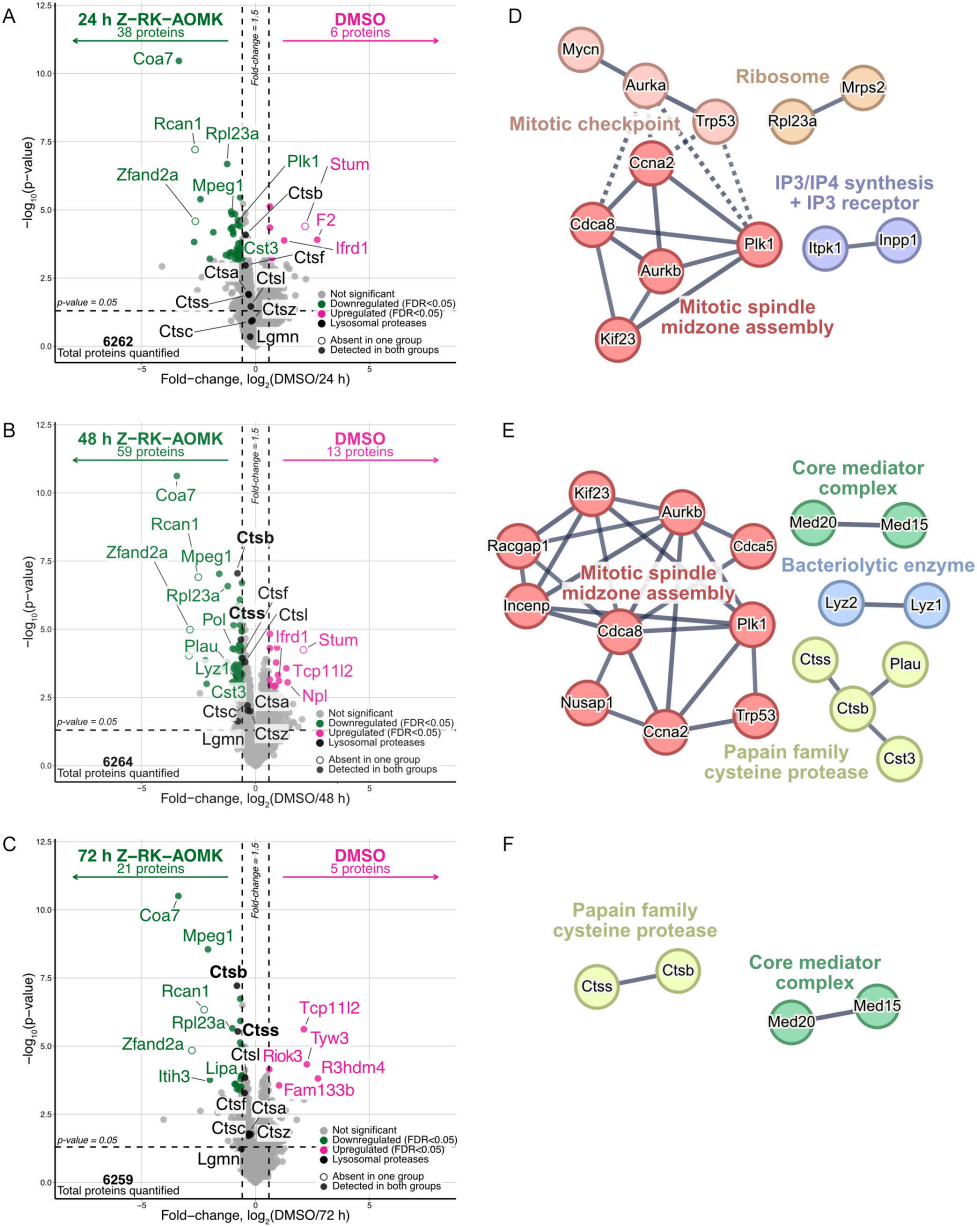
Proteomic analysis of RAW264.7 cells treated with 5 µM Z‐RK‐AOMK across time. (A–C) Scatterplot comparing protein abundance in whole‐cell lysates from DMSO‐ or 5 µM Z‐RK‐AOMK‐treated RAW264.7 cells after 24 h (A), 48 h (B), and 72 h (C). Log_2_ fold‐change in protein abundance is plotted against −Log_10_ *p*‐value (*n* = 4 biological replicates). Significance thresholds were set at ±1.5‐fold change and FDR <0.05. (D–F) STRING network analysis of proteins significantly enriched in Z‐RK‐AOMK‐treated RAW264.7 cells compared to DMSO control at 24 h (D), 48 h (E), and 72 h (F), corresponding to the datasets shown in (A), (B), and (C), respectively. Protein interaction networks were generated using the highest confidence interaction score (0.900), followed by Markov clustering algorithm (MCL) clustering. Disconnected proteins were excluded from the networks.

Protein network analysis using STRING [43] highlighted functional clustering of altered proteins. At 24 and 48 h following Z‐RK‐AOMK treatment, the largest STRING clusters were associated with mitotic regulation and ribosome‐related proteins (Figure 4D,E). By 48 h, additional clusters associated with cysteine proteases (Ctsb, Ctss, Cst3, and Plau) and bacteriolytic enzymes involved in innate immune responses (Lyz1 and Lyz2) became apparent (Figure 4E). At 72 h, the response was more restricted and characterized by a cluster of lysosomal proteases (Ctss and Ctsb) (Figure 4F), which were also present at 48 h.

Cystatin C (Cst3), an endogenous inhibitor of cysteine cathepsins, was increased at 24 and 48 h (Figure 4A,B and Table S4), while Ctss and Ctsb were enriched at 48 and 72 h but not 24 h following Z‐RK‐AOMK treatment (Figure 4A–C and Table S4), suggesting coordinated regulation of protease–inhibitor balance within the lysosomal system [44]. The progressive increase in Ctsb abundance over time with Z‐RK‐AOMK treatment suggests a potential feedback response to compensate for the loss of its enzymatic activity. The increase in Ctss abundance observed here is consistent with the increase seen in the proform and higher molecular weight Ctss species (Figure 2E,H last panel and Table S2). However, the identified Ctss peptides did not provide sufficient information to determine the cleavage site responsible for generation of the higher molecular weight species. Urokinase‐type plasminogen activator (Plau) was increased following 48 h of Z‐RK‐AOMK treatment (Figure 4B,E). Plau has been reported to contribute to pro‐Ctsb activation [45], while Ctsb has also been shown to activate the proenzyme form of Plau [46]. Together, given that Ctsb inhibition has previously been linked to lysosomal dysfunction [47], these proteomics changes likely reflect a compensatory response aimed at restoring lysosomal protease activity and protein homeostasis.

Rcan1 was consistently enriched in Z‐RK‐AOMK‐treated cells across all time points (Table 1, Figure 4A–C, and Table S4), together with early enrichment of inositol phosphate signaling components (Figure 4D), suggesting engagement of calcium‐dependent regulatory pathways consistent with lysosome‐associated signaling in macrophages [48]. Zfand2a was also consistently enriched in Z‐RK‐AOMK treated cells across all time points (Figure 4A–C and Tables 1 and S4). As Zfand2a is associated with proteasome‐mediated clearance of misfolded proteins under proteotoxic stress [49], its enrichment may reflect increased demand on cellular protein quality control pathways following inhibition of Ctsb activity.

**TABLE 1:** Common significant hits from proteomics across the three timepoints.

Uniprot ID	Gene	Protein description	DMSO/24 h^a^	DMSO/48 h^a^	DMSO/72 h^a^
Q921H9	Coa7	Cytochrome c oxidase assembly factor 7	−3.3664912	−3.4566557	−3.3891227
Q9JHG6‐4	Rcan1	Calcipressin‐1	−2.6568796	−2.5135862	−2.2574758
Q9JII7	Zfand2a	AN1‐type zinc finger protein 2A	−2.6439442	−2.8809128	−2.7955554
P62751	Rpl23a	Large ribosomal subunit protein uL23	−1.2455925	−1.22222	−1.0231618
A1L314	Mpeg1	Macrophage‐expressed gene 1 protein	−1.0390437	−1.5959314	−2.0901063
P10400	Pol	Retrovirus‐related Pol polyprotein	−0.9870429	−1.0074096	−0.8176503
P03966	Mycn	N‐myc proto‐oncogene protein	−0.906318	−0.9659345	−0.6682375
P51943	Ccna2	Cyclin‐A2	−0.7881514	−0.7620273	−0.6294723
Q9DBU3	Riok3	Serine/threonine‐protein kinase RIO3	0.62766736	0.62151818	0.59954161

a Log_2_ fold‐change of the protein abundance. Significance thresholds were set at ±1.5‐fold change and adjusted *p‐*value (Benjamini–Hochberg FDR) < 0.05.

### 2.5 Ctsb Inhibition Results in Modest Remodeling of the Cellular N‐Terminome

The proteomic changes observed following sustained Ctsb inhibition suggested remodeling of lysosomal protease networks and broader cellular adaptation (Figure 4 and Table S4). To identify cleavage events altered by Z‐RK‐AOMK treatment, the NICE datasets were re‐searched allowing semispecific N‑terminal cleavage (SEMI_N_TERM) to capture dimethylated peptides resulting from proteolysis. Approximately 90% of the modified peptides were fully dimethylated across all replicates, indicating high dimethylation efficiency (Figure S2B). Of the 52,029 modified peptides detected across all samples, 8219 were N‐terminally dimethylated (Figure S2C). As the assignment of N‐terminal dimethylation events in DIA datasets requires additional scrutiny [50, 51], only peptides containing fully dimethylated internal lysine residues were considered for analysis to avoid potential false positives. This resulted in 917 neo‐N‐termini, defined as fully dimethylated peptides that do not correspond to annotated protein N‐termini (Figure S2C). Neo‐N‐termini peptide intensities were normalized to their corresponding protein abundance before downstream differential abundance analysis [52, 53]. Cleavage events relatively enriched in DMSO‐treated cells were considered Ctsb‐dependent, whether direct or indirect, whereas those enriched upon Z‐RK‐AOMK treatment may reflect compensatory or alternative protease activities associated with perturbation of the cysteine cathepsin network.

Neo‐N‐termini (22, 18, and 22) were significantly enriched in the DMSO‐treated cells after 24, 48, and 72 h treatment with Z‐RK‐AOMK, respectively, while 12, 15, and 15 neo‐N‐termini were enriched in Z‐RK‐AOMK‐treated cells across the time points (Figure 5A–C, and Table S5). Of these, 14 neo‐N‐termini were consistently identified across the time course (Tables 2, 3, and S5). These findings indicate that only a small subset of the detectable N‐terminome is altered following Z‐RK‐AOMK treatment. Consistent with this, sequence motif analysis [55] of differentially enriched neo‐N‐termini did not reveal a robust or biologically interpretable consensus cleavage signature (Figure S3 and Table S6), likely reflecting the limited number of regulated N‐terminal cleavage events and the heterogeneity of contributing proteases.

**FIGURE 5:**
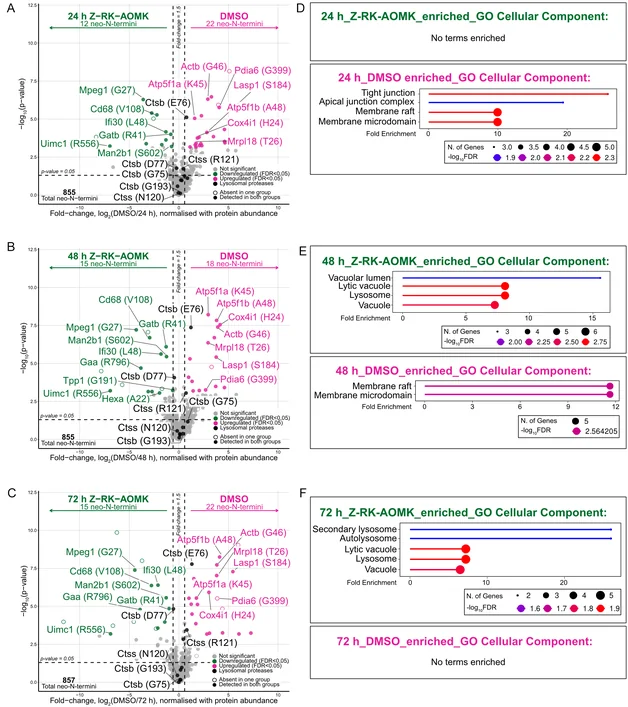
N‐terminomics analysis of RAW264.7 cells treated with 5 µM Z‐RK‐AOMK across time. (A–C) Scatterplots comparing neo‐N‐termini abundance, normalized to protein abundance in whole‐cell lysates from DMSO‐ or 5 µM Z‐RK‐AOMK‐treated RAW264.7 cells after 24 h (A), 48 h (B), and 72 h (C). Log_2_ fold‐change in normalized neo‐N‐termini abundance is plotted against −Log_10_ *p*‐value (*n* = 4 biological replicates). Significance thresholds were set at ±1.5‐fold change and FDR <0.05. (D–F) Gene ontology cellular component enrichment analysis of proteins associated with significantly enriched neo‐N‐termini at 24 h (D), 48 h (E), and 72 h (F), corresponding to the datasets shown in (A), (B), and (C), respectively. Analysis was performed using ShinyGO 0.80 [54]. Proteins associated with all detected neo‐N‐termini were used as the background set for enrichment analysis.

**TABLE 2:** Common neo‐N‐termini enriched in DMSO‐treated cells across three timepoints.

Uniprot ID	Protein description	Genes	Cleavage site	Region/domain cleaved
P19783	Cytochrome c oxidase subunit 4 isoform 1, mitochondrial	Cox4i1	H24	Transit peptide (1–22)
Q03265	ATP synthase F(1) complex subunit alpha, mitochondrial	Atp5f1a	K45	Transit peptide (1–43)
P56480	ATP synthase F(1) complex catalytic subunit beta, mitochondrial	Atp5f1b	A48	Transit peptide (1–46)
Q9CQL5	Large ribosomal subunit protein uL18m	Mrpl18	T26	Transit peptide (1–38)
Q922R8	Protein disulfide‐isomerase A6	Pdia6	G399	Disordered region (399–440)
Q61792	LIM and SH3 domain protein 1	Lasp1	S184	Disordered region (123–207)
P10605	Cathepsin B	Ctsb	E76	Propeptide (18–79)
P60710	Actin, cytoplasmic 1	Actb	G46	Whole protein (1–375)

**TABLE 3:** Common neo‐N‐termini enriched in Z‐RK‐AOMK‐treated cells across three time points.

Uniprot ID	Protein description	Genes	Cleavage site	Region/domain cleaved
Q5U5Q9	BRCA1‐A complex subunit RAP80	Uimc1	R556	Zinc‐finger‐like region (513–590)
A1L314	Macrophage‐expressed gene 1 protein	Mpeg1	G27	Non‐cytoplasmic domain (20–653)
P31996	Macrosialin	Cd68	V108	Mucin‐like region (21–109)
O09159	Lysosomal alpha‐mannosidase	Man2b1	S602	Galactose mutarotase‐like domain (475–1005)
Q9ESY9	Gamma‐interferon‐inducible lysosomal thiol reductase	Ifi30	L48	Propeptide (27–54)
Q99JT1	Glutamyl‐tRNA(Gln) amidotransferase subunit B, mitochondrial	Gatb	R41	Disordered region (21–58)

Notably, Ctsb itself was cleaved at residue E76 with higher abundance in DMSO‐treated cells across all time points (Figure ‍5A–‍C and Tables 2 and S5), corresponding to a site located at the boundary of its propeptide region (residues 18–79). This observation is consistent with the known autocatalytic activation of Ctsb as reported previously [39, 56, 57]. At 48 and 72 h, a neo‐N‐terminus corresponding to Ctss cleavage at R121, which is at the end of its propeptide region (residues 18–122), was also enriched in the DMSO‐treated cells (Figure 5B,C and Table S5). We did not detect neo‐N‐termini corresponding to cleavage sites upstream of N120 that would support the higher molecular weight Ctss species observed by immunoblotting (Figures 1D, 2A,E,F, and 3A). Together, these findings suggest that Z‐RK‐AOMK treatment reduces propeptide processing of Ctsb and Ctss.

Eight neo‐N‐termini enriched in DMSO‐treated cells were conserved across all three time points (Figure 5A–C and Tables 2 and S5). Besides Ctsb, this core set included mitochondrial proteins (Cox4i1, Atp5f1a, Atp5f1b, Mrpl18), cytoskeletal proteins (Actb, Lasp1), and the endoplasmic reticulum protein Pdia6involving in unfolded protein response [58]. The mitochondrial cleavage sites were consistent with removal of mitochondrial targeting sequences during protein import into the mitochondrial matrix [59], indicating that many of the detected neo‐N‐termini may represent constitutive mitochondrial processing events rather than direct Ctsb substrates.

Gene ontology analysis revealed that neo‐N‐termini enriched in DMSO‐treated cells were associated with membrane raft and membrane microdomain components (Figure 5D,E, bottom panel). In contrast, neo‐N‐termini enriched following Z‐RK‐AOMK treatment showed significant association with lysosomal cellular components (Figure 5E,F, top panel). Among these were cleavage events derived from lysosomal and endolysosomal proteins including Mpeg1, Ifi30, Cd68, and Man2b1, which were consistently enriched across all time points (Figure 5A–C and Tables 3 and S5). The persistence of this group of proteins, all of which are associated with lysosomal or phagolysosomal function in macrophages, suggests that inhibition of Ctsb may preferentially affect proteolytic processing within the endolysosomal system.

Additional neo‐N‐termini from lysosomal hydrolases were enriched in Z‐RK‐AOMK treated cells at later timepoints, including Hexa (A22) cleaved following removal of its signal peptide (1–22) (Figure 5B), Tpp1 (G191) cleaved near the end of its propeptide region (20–194) (Figure 5B and Table S5), and Gaa (R796) cleaved within its glycosyl hydrolase domain (705–821) (Figure 5B,C and Table S5).

### 2.6 Ctsb Mainly Functions as a Dipeptidyl Carboxypeptidase in RAW264.7 Cells

Although N‐terminomics analysis revealed modest remodeling of the protein cleavage landscape following Ctsb inhibition, Ctsb is known to function predominantly as a dipeptidyl carboxypeptidase rather than an endopeptidase [10]. C‐terminal cleavage events generated by Ctsb are therefore expected to be reduced following Z‐RK‐AOMK treatment. We therefore examined changes in the cellular C‐terminome by re‐searching the mass spectrometry data using semi‐specific trypsin parameters (SEMI), enabling detection of non‐tryptic peptides at both termini [60] (Figure S2E).

As primary amines were dimethylated prior to digestion, lysine residues were chemically blocked, restricting trypsin cleavage primarily to arginine residues [23, 61, 62]. Peptides with C‐termini consistent with trypsin‐derived cleavage at arginine residues, and those corresponding to annotated native protein C‐termini, were excluded (Figure S2E). The remaining 1396 peptides were retained as neo‐C‐termini (Figure S2E) and normalized to their corresponding protein abundance prior to downstream differential analysis.

In contrast to the N‐terminome, inhibition of Ctsb with Z‐‍RK‐AOMK produced a pronounced alteration in the cellular C‐‍terminome. Neo‐C‐termini (63, 67, and 61) were significantly enriched in DMSO‐treated cells after 24, 48, and 72 h treatment with Z‐RK‐AOMK, respectively, indicating that these cleavage events are associated with Ctsb activity under basal conditions (Figure 6A–C and Table S7). Of these, 54 DMSO‐enriched neo‐C‐termini were shared across conditions, suggesting that a core set of C‐terminal processing events is consistently associated with Ctsb activity.

**FIGURE 6:**
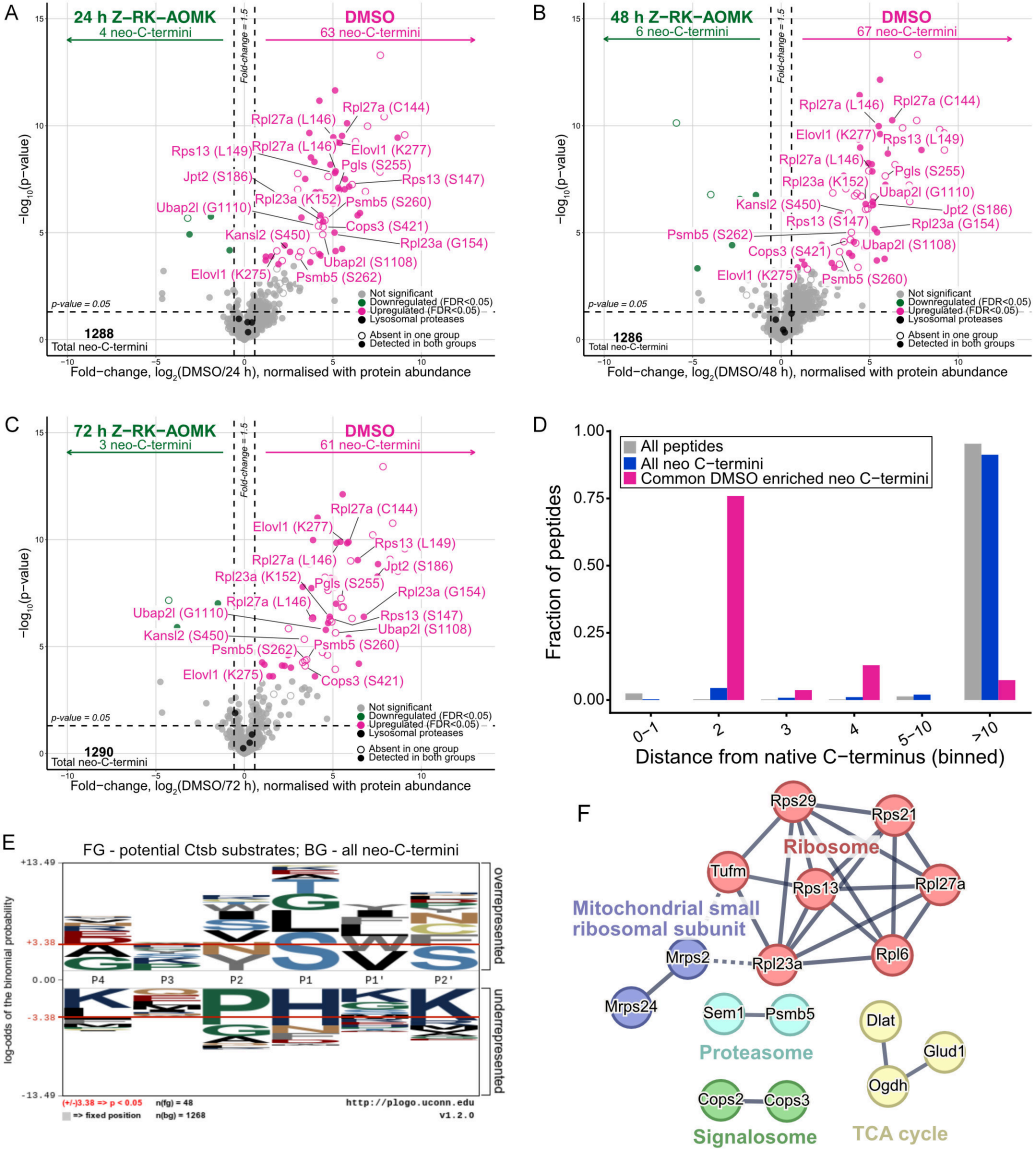
C‐terminomics analysis of RAW264.7 cells treated with 5 µM Z‐RK‐AOMK across time. (A–C) Scatterplots comparing neo‐C‐termini abundance, normalized to protein abundance in whole‐cell lysates from DMSO‐ or 5 µM Z‐RK‐AOMK‐treated RAW264.7 cells after 24 h (A), 48 h (B), and 72 h (C). Log_2_ fold‐change in normalized neo‐C‐termini abundance is plotted against −Log_10_ *p*‐value (*n* = 4 biological replicates). Significance thresholds were set at ±1.5‐fold change and FDR <0.05. (D) Distribution of distances between neo‐C‐termini cleavage sites and the native protein C‐terminus. The bar chart shows the fraction of peptides at each distance for all detected peptides, all neo‐C‐termini, and neo‐C‐termini significantly enriched in DMSO‐treated cells across all three time points (A–C). (E) pLogo analysis of the P4–P2′ flanking sequences of neo‐C‐termini consistently and significantly enriched in DMSO‐treated cells, with cleavage events occurring two or four residues from the native protein C‐terminus. The P4–P2’ flanking sequences of all identified neo‐C‐termini were used as the background. Serine (S) at the P1 position was significantly enriched (16.7%, log‐odds score = 4.9, *p* = 0.001). (F) STRING network analysis of proteins associated with neo‐C‐termini consistently significantly enriched in DMSO‐treated cells with cleavage sites located two or four residues from the native protein C‐terminus. Protein interaction networks were generated using the highest confidence interaction score (0.900), followed by Markov clustering algorithm (MCL) clustering. Disconnected proteins were excluded from the network.

Given the established role of Ctsb as a dipeptidyl carboxypeptidase [10], we next examined the positional relationship between cleavage sites and native protein C‐termini (Figure 6D). Strikingly, 41 of the 54 common DMSO‐enriched neo‐C‐termini (~76%) were generated by cleavage exactly two residues upstream of the native C‐terminus, with a further seven cleavage sites (~13%) occurring four residues upstream (Figure 6D, and Table S8). Overall, 50 of 54 sites mapped within four residues of the native C‐terminus, whereas only four cleavage sites occurred more than 10 residues upstream of their native C‐terminus. This distribution differed significantly from that observed for all peptides or all neo‐C‐termini, which have their C‐terminal cleavage sites predominantly located more than ten residues upstream of their native protein C‐termini (Figure 6D, 95% and 91%, respectively). These data are consistent with sequential C‐terminal trimming via dipeptidyl carboxypeptidase activity [10, 63] and indicate that many of the observed cleavage events may reflect Ctsb‐mediated C‐terminal processing.

The neo‐C‐termini generated by cleavage at two or four residues from the native C‐terminus (Figure 6D, and Table S8) were analyzed using pLogo [55] to identify sequence preferences surrounding the cleavage sites (Figure 6E and Table S8). Serine was the only residue significantly enriched at the P1 position, occurring with a frequency of 16.7% (log‐odds score = 4.9, *p‐‍*value = 0.001) (Figure 6E). While human CTSB has been reported to prefer basic residues such as arginine and lysine at P1 using peptide libraries [10, 64, 65, 66], Cotrin et al. [66] also showed that smaller side‐chain residues, including serine, are tolerated by CTSB at P1. The enrichment observed here may reflect Ctsb substrate preferences within the endogenous mouse proteome, although interpretation is limited by the inability to distinguish endogenous cleavage events generating C‐terminal arginine residues from trypsin‐derived peptides.

Gene ontology analysis of potential Ctsb‐associated substrates (DMSO‐enriched neo‐C‐termini generated by cleavage two or four residues upstream of native protein C‐termini)did not identify significantly enriched biological processes when all neo‐C‐termini were used as the background set. To investigate potential functional relationships, STRING network analysis [43] was performed. The largest interaction network contained ribosomal and translation‐associated proteins, suggesting enrichment of Ctsb‐associated C‐terminal cleavage events within proteins involved in translation (Figure 6F). Several smaller interaction clusters were also identified, including proteins associated with the proteasome (Psmb5 and Sem1), COP9 signalosome‐related proteins (Cops2 and Cops3), and the tricarboxylic acid (TCA) cycle (Glud1, Ogdh, and Dlat) (Figure 6F). Together, these observations suggest that Ctsb‐associated C‐terminal processing is enriched within multiple functional protein networks involved in protein homeostasis and cellular metabolism.

To further narrow candidate substrates for Ctsb‐associated cleavage, we focused on neo‐C‐termini generated by cleavage at serine residues located two or four residues upstream of the native protein C‐terminus and examined the abundance of the corresponding native C‐terminal peptides (Figure ‍7). This analysis identified six candidate proteins: Cops3, Jpt2, Kansl2, Pgls, Psmb5, and Ubap2l. Notably, four of these six proteins possess intrinsically disordered C‐terminal regions at or near the identified cleavage sites (Table 4), suggesting that local structural flexibility may facilitate accessibility to Ctsb‐associated processing [67].

**FIGURE 7:**
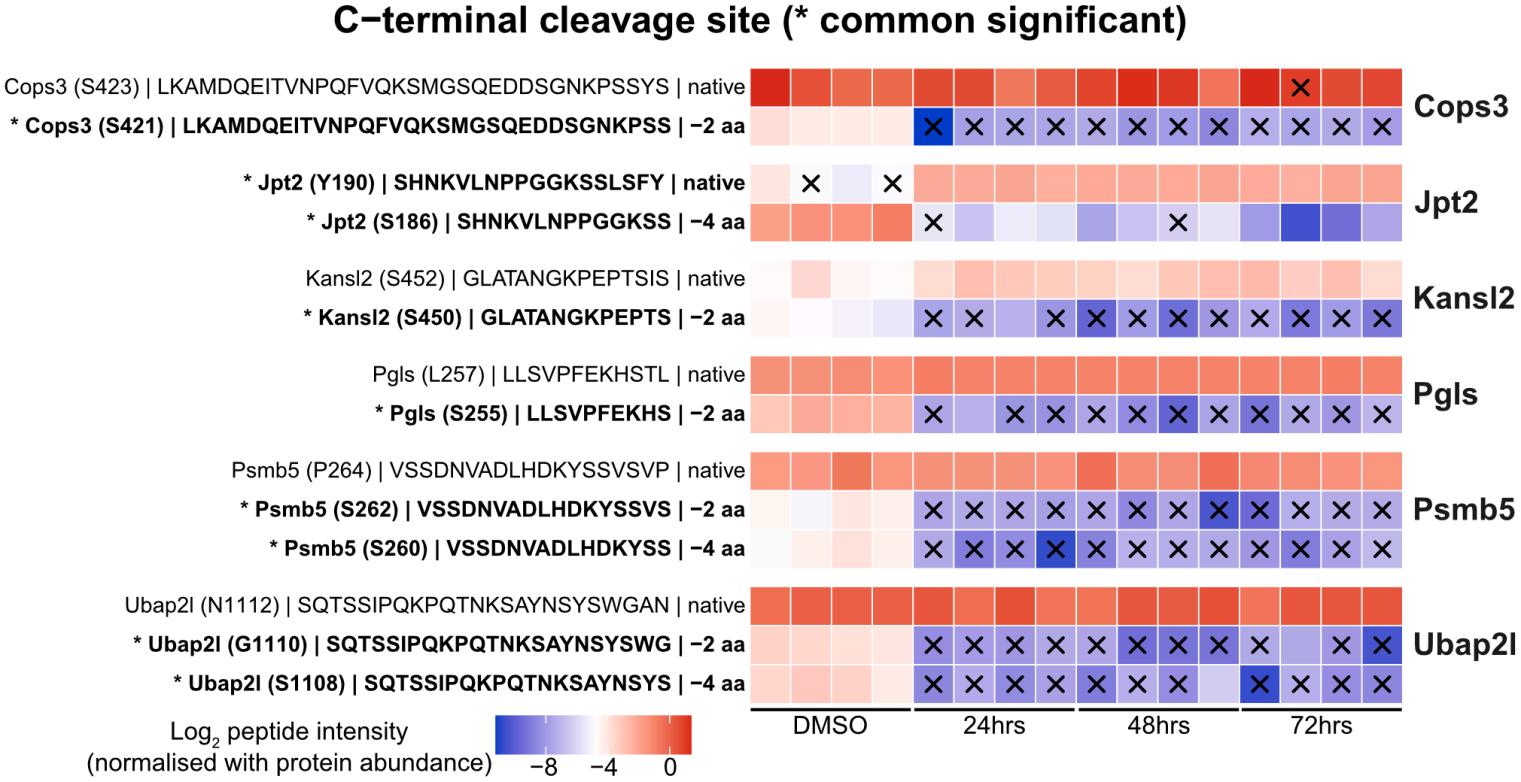
Putative Ctsb substrates. Heatmap showing Log_2_ peptide abundance normalized to protein abundance for neo‐C‐termini consistently significantly enriched in DMSO‐treated cells across all three time points. Included neo‐C‐termini had cleavage sites located two or four residues from the native protein C‐terminus and were derived from proteins in which at least one neo‐C‐terminus was cleaved at serine (P1 position). Corresponding native protein C‐termini are also shown. Replicates with imputed values are indicated by a cross (X) within the corresponding box (meaning peptides were not detected in the indicated samples). C‐termini exhibiting a significant difference between DMSO‐ and Z‐RK‐AOMK‐treated cells (±1.5‐fold change and FDR < 0.05) are shown in bold and marked with asterisk.

**TABLE 4:** Candidate substrates of Ctsb dipeptidyl carboxypeptidase activity.

Uniprot ID	Protein description	Genes	Cleavage site	Distance from C‐terminus	Region/domain cleaved
O88543	COP9 signalosome complex subunit 3	Cops3	S421	2	Disordered region (402–423)
Q6PGH2	Jupiter microtubule associated homolog 2	Jpt2	S186	4	Disordered region (1–190)
Q8BQR4‐2	Isoform 2 of KAT8 regulatory NSL complex subunit 2	Kansl2	S450	2	Disordered region (406–452)
Q9CQ60	6‐Phosphogluconolactonase	Pgls	S255	2	C‐terminus
O55234	Proteasome subunit beta type‐5	Psmb5	S262	2	C‐terminus
			S260	4	
Q80X50‐5	Isoform 5 of ubiquitin‐associated protein 2‐like	Ubap2l	G1110	2	Disordered region (1065–1112)
			S1108	4	

With the exception of Jpt2, the abundance of the native C‐‍terminal peptides did not differ significantly between DMSO‐ and Z‐RK‐AOMK‐treated cells, indicating that only a fraction of the total pool of each protein is cleaved in naïve cells (Figure 7). In contrast, Jpt2 showed significantly reduced abundance of the native C‐terminal peptide in DMSO‐treated cells, together with a corresponding increase in a neo‐C‐terminus generated by cleavage at S186, located four residues upstream of the native C‐terminus (Figure 7). No corresponding cleavage product with two residues upstream of the native C‐terminus was detected for Jpt2 (Figure 7 and Table 4). Taken together, these data suggest that most of Jpt2 is subject to two sequential dipeptidyl carboxypeptidase events in naïve RAW264.7 cells.

Psmb5 and Ubap2l each contained neo‐C‐termini cleaved at both two and four residues upstream of native C‐terminus that were significantly enriched in DMSO‐treated cells (Figure 7 and Table 4). The detection of both cleavage products is consistent with progressive dipeptidyl carboxypeptidase activity [10, 63], supporting these proteins as candidate Ctsb‐associated substrates. Notably, both Psmb5 cleavage sites occur at serine residues (Figure 7 and Table 4), consistent with the P1 preference identified by pLogo analysis (Figure 6E). Both Psmb5 and Ubap2l are associated with protein homeostasis pathways (Figure 7 and Table 4). Psmb5 is a catalytic subunit of the 20S proteasome [68], while Ubap2l has been implicated in protein quality‐control pathways through its role in stress granule assembly [69]. In addition, Cops3, which is a component of the COP9 signalosome that regulates the ubiquitin‐proteasome system [70], also exhibited a C‐‍terminal cleavage event enriched in DMSO‐treated cells (Figure 7 and Table 4). Together, these observations suggest that Ctsb‐associated C‐terminal processing may involve proteins linked to proteostasis.

As lysosomal degradation contributes to proteasome turnover through a selective autophagic pathway termed proteaphagy [71], some of the identified cleavage events may reflect physiological processing associated with protein complex turnover. Dysregulation of both cathepsin B and proteasome activity has been reported in dementia with Lewy bodies, a neurodegenerative disorder characterized by protein aggregation [72]. Further experiments will be required to determine whether these proteins represent direct Ctsb substrates and whether Ctsb contributes to proteasome turnover under basal conditions.

### 2.7 Synthesis of a New Activity Probe, BODIPY‐RK‐AOMK

While Z‐RK‐AOMK enables inhibition of Ctsb activity, it does not provide information regarding the localization or spatial distribution of active Ctsb within cells. To complement the inhibitor studies, a fluorescent activity‐based probe was generated by conjugating a BODIPY‐FL fluorophore in place of the carbobenzoxy capping group in the Z‐RK‐AOMK scaffold (Figure 8A).

**FIGURE 8:**
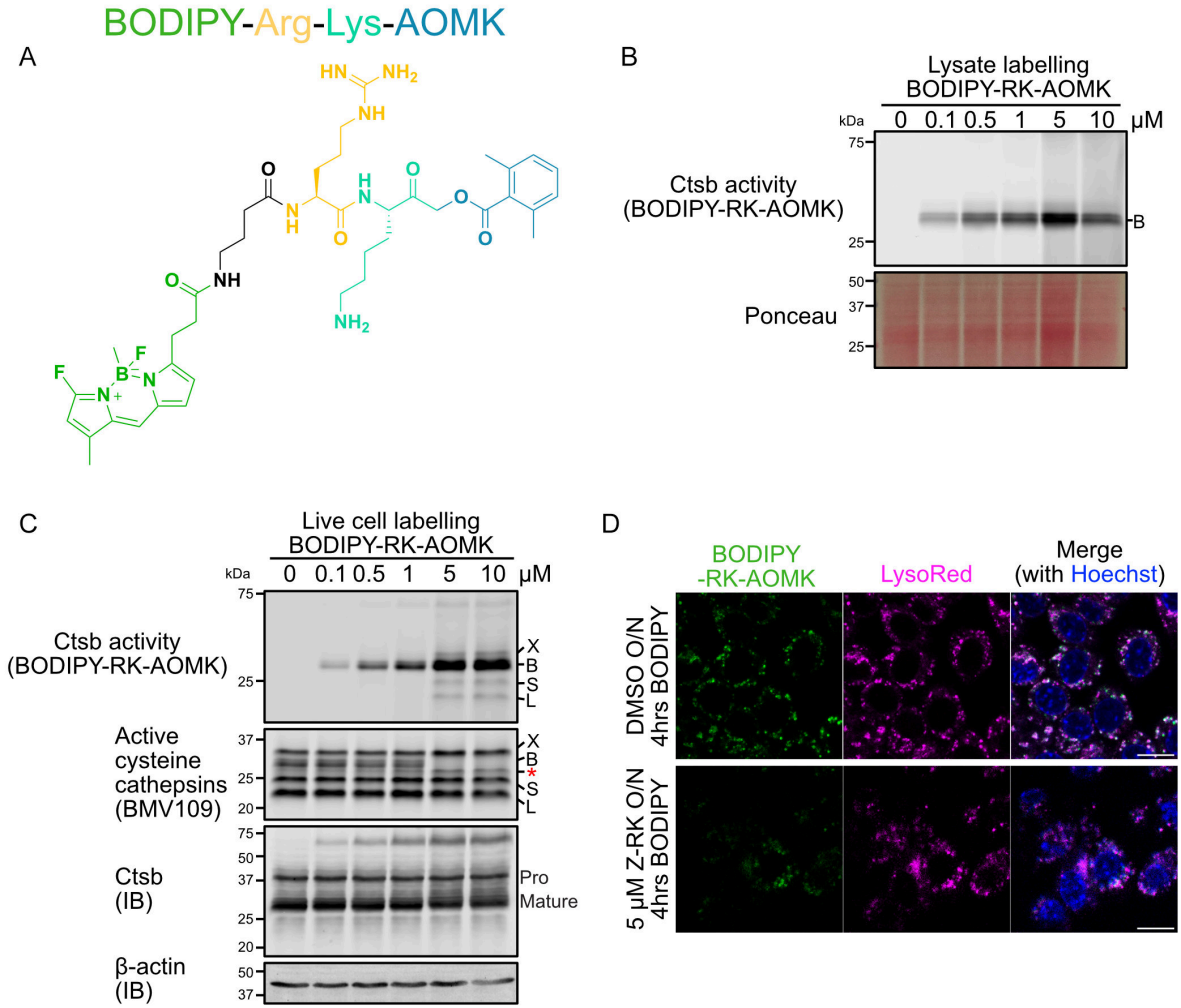
BODIPY‐RK‐AOMK labels intracellular Ctsb with limited off‐target labeling of Ctsx. (A) Chemical structure of BODIPY‐RK‐AOMK. (B) RAW264.7 cell lysates in citrate lysis buffer were labeled with increasing concentrations of BODIPY‐RK‐AOMK (0–10 µM). The labeled cell lysates were analyzed by SDS–PAGE followed by in‐gel Cy2 fluorescence imaging. (C) RAW264.7 cells were live‐labeled with increasing concentrations of BODIPY‐RK‐AOMK (0–10 µM) for 4 h. Residual active cysteine cathepsins were then labeled with 1 µM BMV109 for 4 h. Cells were harvested, lysed in PBS containing 0.1% Triton X‐100, and analyzed by SDS–PAGE followed by in‐gel Cy2 and Cy5 fluorescence imaging and immunoblotting. Corresponding raw images are provided in the Supporting Information. (D) Immunofluorescence imaging of RAW264.7 cells labeled with BODIPY‐RK‐AOMK (green). Cells were pre‐treated with either 0.1% DMSO (top panel) or 5 µM Z‐RK‐AOMK (bottom panel) for 16 h, followed by live labeling with 1 µM BODIPY‐RK‐AOMK for 4 h. Cells were washed with PBS and incubated in fresh medium for 1 h to remove unbound probe, then stained with 50 nM LysoTracker Red (magenta) and Hoechst 33258 (blue) for live‐cell imaging. Images were acquired using Zeiss Elyra/LSM880. Scale bar, 10 µm.

As Z‐RK‐AOMK has been reported to preferentially inhibit Ctsb under neutral pH conditions [11], incorporation of a fluorophore enables visualization of active Ctsb and provides a means to assess its intracellular localization. The acyloxymethyl ketone (AOMK) warhead irreversibly reacts with the catalytic cysteine residue of active Ctsb, allowing the resulting BODIPY‐RK‐AOMK probe to function both as an inhibitor and an activity‐based probe for direct detection of active Ctsb in cells.

BODIPY‐RK‐AOMK probe was synthesized using a modified version of a previously reported procedure [11]. Briefly, synthesis commenced with the bromomethyl ketone derivative of Fmoc‐Lys‐OH **1**, which was acylated with 2,6‐dimethylbenzoic acid **2** in the presence of potassium fluoride to afford the ketomethyl ether intermediate 3 (Scheme 1). Subsequent removal of the Fmoc protecting group using DBU in dichloromethane (DCM) generated the corresponding free amine, which was coupled to Fmoc‐Arg(Pbf)‐OH using HATU and Hünig’s base to yield the C‐terminally modified dipeptide **4**. The Fmoc group of **4** was then removed with DBU, and the resulting amine intermediate was coupled to BODIPY‐FL‐NH‐butanoic acid **5** using HATU and DIPEA to produce the N‐terminally labeled dipeptide **6**. Finally, global deprotection was achieved by treatment of **6** with trifluoroacetic acid (TFA) in the presence of triisopropylsilane (TIPS) in DCM, resulting in removal of the Pbf and Boc protecting groups and furnishing the fluorogenic probe WEHI‐237, subsequently named BODIPY‐RK‐AOMK.

**SCHEME 1:**
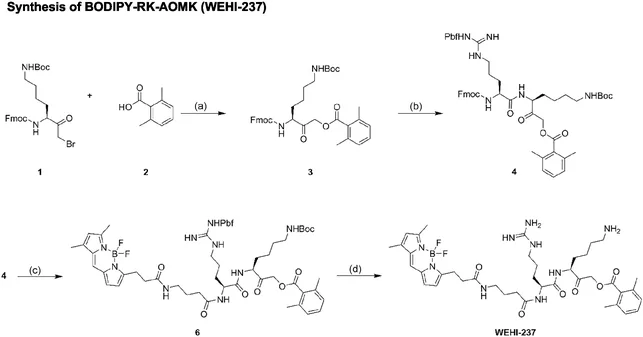
Reaction conditions: (a) KF, 2,6‐dimethylbenzoic acid, DMF, r.t. (b) (i) DCM and DBU and (ii) Fmoc‐Arg(Pbf)‐OH, HATU, DIPEA, DCM, r.t. (c) (i) DCM and DBU and (ii) **5**, HATU, DIPEA, DCM, r.t. (d) TFA, TIPS, DCM, r.t.

### 2.8 BODIPY‐RK‐AOMK Labels Ctsb and Shows Limited Cross‐Reactivity With Other Cathepsins Under Live‐Cell Conditions

Titration of BODIPY‐RK‐AOMK in RAW264.7 cell lysates showed concentration‐dependent labeling of active Ctsb, with detectable labeling observed at 0.1 µM (Figure 8B). In cell lysates, labeling remained predominantly Ctsb‐specific up to 10 µM (Figure 8B). Live‐cell labeling with BODIPY‐RK‐AOMK followed by BMV109 labeling showed near‐complete labeling of the active Ctsb pool at 5 µM, as indicated by the loss of subsequent BMV109 labeling (Figure 8C). However, at higher concentrations (5 and 10 µM), BODIPY‐RK‐AOMK also labelled Ctsx, Ctss, and Ctsl to a small extent under live‐cell conditions (Figure 8C). As observed for the parent compound Z‐RK‐AOMK (Figure 1B), BODIPY‐RK‐AOMK also altered the activity profiles of other cysteine cathepsins, as revealed by BMV109 labeling (Figure 8C).

To visualize the localization of active Ctsb, cells were labeled with 1 µM BODIPY‐RK‐AOMK, a concentration that minimized detectable Ctsx, Ctss, and Ctsl labeling under live‐cell conditions (Figure 8C). Live‐cell imaging showed substantial colocalization of BODIPY‐RK‐AOMK signal with LysoTracker Red (Figure 8D, top panel), consistent with predominant endolysosomal localization of active Ctsb in healthy cells [8]. Residual fluorescence persisted following pretreatment with 5 µM Z‐RK‐AOMK prior to probe incubation (Figure 8D, bottom panel). This residual signal may reflect a combination of low‐level off‐target probe retention, including labeling of other cathepsins, ornon‐specific intracellular accumulation of the hydrophobic BODIPY fluorophore, which can contribute to background fluorescence [73]. The bright signal concentrated in lysosomes may mask more diffuse signal in other cellular locations, and it is also possible that BODIPY‐RK‐AOMK traffics to different intracellular locations than Z‐RK‐AOMK; thus, it remains difficult to draw definitive conclusions about Ctsb activity in neutral compartments.

## 3 Conclusion

This study provides a comprehensive characterization of Z‐RK‐AOMK in RAW264.7 cells. While Z‐RK‐AOMK effectively inhibited intracellular Ctsb activity, it also increased Ctsx activity, reduced Ctsl activity, the latter of which induced the accumulation of a glycosylated higher molecular weight species of active Ctss. These findings demonstrate that Z‐RK‐AOMK perturbs the broader cysteine cathepsin network and reveal an unexpected, most likely Ctsl‐dependent, effect on Ctss maturation.

Despite these changes in cysteine cathepsin activity, global proteomic and N‐terminomic analyses revealed surprisingly limited effects on protein abundance and N‐terminal cleavage events following Z‐RK‐AOMK treatment. These findings suggest that basal cellular proteolytic networks are largely buffered against complete loss of Ctsb activity.

In contrast, C‐terminal degradomics uncovered a highly reproducible cleavage signature positioned two and four residues from native protein C‐termini. This provides strong evidence supporting dipeptidyl carboxypeptidase activity as the dominant mode of Ctsb function in cells and highlights C‐‍terminome profiling as a powerful approach for resolving Ctsb‐dependent proteolysis.

Collectively, these findings refine the functional view of Ctsb in macrophages, positioning it as a regulated protease with selective processing activity rather than a broadly degradative protease under basal conditions. Finally, the development of the fluorescent activity‐based probe BODIPY‐RK‐AOMK provides a complementary tool for interrogating active Ctsb in situ and establishes a platform for future studies investigating the spatial regulation and biological functions of Ctsb.

## 4 Materials and Methods

### 4.1 Cell Culture

RAW264.7 cells (an immortalized mouse monocyte/macrophage‐like cell line; ATCC TIB‐71; RRID:CVCL_0493) were cultured in Dulbecco’s Modified Eagle Medium (DMEM; high glucose, Gibco #11995073) supplemented with 10% [*v/v*] fetal bovine serum (FBS; CellSera #AU‐FBS/PG) and 1% [*v/v*] penicillin‐streptomycin (100 U/mL; Thermo Fisher Scientific #15140122). Cells were incubated at 37 °C in a humidified incubator with 5% CO_2_. When required, cells were detached from tissue culture flasks or plates using sterile cell scrapers.

### 4.2 Small Molecule Inhibitor, Z‐RK‐AOMK

RAW264.7 cells were seeded onto tissue culture plates and allowed to adhere overnight prior to treatment with Z‐RK‐AOMK (synthesized in‐house) [11, 12]. Z‐RK‐AOMK was diluted to the required concentration in DMEM with a final 0.1% [*v/v*] DMSO and applied to the cells. Cells were incubated with Z‐RK‐AOMK‐ containing medium for 16 h before activity‐based probe labeling (Section 4.3) for SDS–PAGE analysis (Section 4.5), or for 24, 48, or 72 h before NICE analysis (Section 4.8). For treatment longer than 24 h, fresh medium containing Z‐RK‐AOMK was replaced every 24 h.

### 4.3 Activity‐Based Probe Live‐Cell Labeling

All activity‐based probes were prepared in DMSO. Following 16 h inhibition with Z‐RK‐AOMK (Section 4.2), cells were incubated in medium containing 1 µM (final 0.1% [*v/v*] DMSO) of the following probes for 4 h before harvesting: BMV109 [27] (labels Ctsx, Ctsb, Ctss, Ctsl), DCG‐04 [33] (labels Ctsx, Ctsb, Ctss, Ctsl), and sCy5‐Nle‐CY [32] (labels Ctsx and Ctss). For BODIPY‐RK‐AOMK labeling, cells were incubated with the indicated concentration of probe in medium (final 0.1% [*v/v*] DMSO) for 4 h. Where indicated, cells were pre‐treated with 5 µM CA‐074 (Sigma 205530) or Cathepsin L inhibitor I (Z‐FF‐FMK; Sigma219421).

Following live‐cell labeling, cells were harvested, washed with PBS, and lysed in PBS containing 0.1% TX‐100 for 30 min on ice. Cell lysates were clarified by centrifugation (21,000 × *g*, 5 min, 4 °C) and protein concentrations were determined using Pierce BCA protein assay kits (Thermo Fisher Scientific #23223, #23224) for downstream analysis (Sections 4.4, 4.5, and 4.7).

### 4.4 Immunoprecipitation for SDS–PAGE Analysis

Following protein quantification (Section 4.3), 40 µg of protein were aliquoted per input and pulldown samples in a volume of 10 µL lysis buffer. Samples were denatured by addition of 5x Laemmli sample buffer (final 1x; 50% glycerol (Sigma‐Aldrich), 250 mM Tris‐Cl (Sigma‐Aldrich), pH 6.8, 10% sodium dodecyl sulfate (SDS; VWR LifeSciences), 0.04% bromophenol blue (Sigma‐Aldrich), 6.25% β‐mercaptoethanol (Sigma‐Aldrich)), and boiled at 95 °C for 5 min.

For pulldown samples, 500 µL of immunoprecipitation buffer (PBS, pH 7.4, 0.5% NP‐40, 1 mM EDTA) was added on ice, followed by antibodies against proteins of interest: 10 µL goat anti‐mouse Ctsx (R&D #AF1033), 10 µL goat anti‐mouse Ctsb (R&D #AF965), 20 µL goat anti‐human Ctss (R&D #AF1183), and 10 µL goat anti‐mouse Ctsl (R&D #AF1515). Subsequently, 40 µL of equilibrated Protein A/G PLUS‐Agarose bead slurry (Santa Cruz #sc‐2003) was added, and samples were incubated overnight at 4 °C with end‐over‐end rotation.

Agarose beads were pelleted by brief centrifugation and washed four times with immunoprecipitation buffer, followed by a final wash with 0.9% NaCl. Proteins bound to the beads were eluted by addition of 20 µL of 2x Laemmli sample buffer and boiling at 95 °C for 5 min before SDS‐PAGE analysis (Section 4.5).

### 4.5 SDS–PAGE, In‐Gel Fluorescence, and Immunoblots

SDS‐PAGE was used for the electrophoresis of denatured protein samples. Gels were prepared in‐house using a 15% resolving gel (15% acrylamide (Bio‐Rad #1610158), 0.375 M Tris, pH 8.8, 0.1% SDS) overlaid with a 4% stacking gel (4% acrylamide, 0.125 M Tris, pH 6.8, 0.1% SDS). Resolving and stacking gels were polymerized by the addition of tetramethylethylenediamine (TEMED; Sigma‐Aldrich #T22500) and 10% ammonium persulfate (APS; Sigma‐Aldrich). Samples prepared in Laemmli sample buffer (Section 4.4) were boiled and loaded onto SDS–PAGE gels alongside Precision Plus Protein Dual Color Standards (Bio‐Rad #1610374). Electrophoresis was performed using Laemmli running buffer (25 mM Tris, 192 mM glycine, 0.1% SDS). For in‐gel fluorescence imaging, gels were scanned after electrophoresis for Cy5 (BMV109) or Cy2 (BODIPY‐RK‐AOMK) fluorescence using an Amersham Typhoon scanner (GE healthcare). Proteins were transferred to nitrocellulose membranes using Trans‐Blot Turbo Transfer System (Bio‐Rad) according to the manufacturer’s instructions. Membranes were than stained with 0.1% Ponceau S (Sigma‐Aldrich #P3504‐10G), 5% acetic acid before being incubated overnight at 4 °C with primary antibodies diluted 1:1 in Intercept Blocking Buffer (LI‐COR) and PBS containing 0.05% TWEEN‐20 (PBST; Sigma‐Aldrich). Primary antibodies used in this study included goat anti‐mouse Ctsx (1:1000, R&D #AF1033), goat anti‐mouse Ctsb (1:1000, R&D #AF965), goat anti‐human Ctss (1:500, R&D #AF1183), goat anti‐mouse Ctsl (1:1000, R&D #AF1515), and rabbit anti‐β‐actin (1:10,000, Thermo Fisher Scientific #MA5‐15 739).

Following primary antibody incubation, membranes were washed three times with PBST and incubated for 1 h at RT with the appropriate secondary antibodies, including donkey anti‐goat IgG conjugated to IRDye 800CW (1:10,000, LI‐COR #92632214) and donkey anti‐rabbit IgG‐HRP (1:10,000, Invitrogen #A16035). In addition to antibody‐based detection, streptavidin conjugated to DyLight 800 (1:10,000, overnight incubation at 4 °C, Invitrogen #21851) was used to detect DCG‐04 following SDS‐PAGE and western transfer.

Membranes were subsequently washed three times with PBST. IRDye 800CW fluorescence was detected using an Amersham Typhoon scanner (GE healthcare), whereas HRP‐conjugated blots were visualized using Clarity Western ECL Substrate (Bio‐Rad) and imaged on a ChemiDoc Imaging System (Bio‐Rad).

### 4.6 Live‐Cell Imaging with BODIPY‐RK‐AOMK

RAW264.7 cells were seeded onto µ‐Slide 8‐well ibidi chamber slides (ibidi #80826) and allowed to adhere in a humidified incubator at 37 °C with 5% CO_2_. Cells were then treated with either 0.1% DMSO or 5 µM Z‐RK‐AOMK for 16 h. Following treatment, cells were live‐labeled with 1 µM BODIPY‐RK‐AOMK for 4 h, washed three times with PBS, and incubated in fresh culture medium for 1 h to allow unbound probe to diffuse out of the cells. Cells were subsequently labeled with 50 nM LysoTracker Red (Invitrogen #L7528) for 1 h, followed by nuclear staining with 10 µg/mL Hoechst 33 258 (Sigma‐Aldrich #14530) for 5 min. Cells were washed three times with PBS and imaged in fresh Opti‐MEM medium (Gibco #31985070) using a Zeiss Elyra LSM880 microscope (Carl Zeiss MicroImaging) equipped with a Plan‐Apochromat 63×/1.40‐NA oil immersion objective. Images were processed using Fiji software [74].

### 4.7 Affinity Purification of DCG‐04‐Labeled Proteins for Mass Spectrometry

Following protein quantification of DCG‐04 live‐labeled cell lysates (Section 4.3), 1 mg of protein per sample was adjusted to a final volume of 500 µL. DCG‐04‐labeled proteins were enriched by adding 15 µL of equilibrated Pierce high‐capacity streptavidin agarose beads (Thermo Fisher Scientific #20361) to each sample, followed by overnight incubation at 4 °C with end‐over‐end rotation.

Streptavidin agarose beads were washed using Pierce Spin Columns (Thermo Fisher Scientific #69705) fitted with a large frit at the bottom of the resin bed to minimize bead loss. Prior to sample loading, columns were equilibrated with 400 µL PBS containing 0.1% TX‐100 by centrifugation (100×*g*, 1 min, 4 °C). Samples were then loaded onto the columns and centrifuged (100×*g*, 1 min, 4 °C). Beads were washed three times with PBS containing 0.1% TX‐100, followed by three washes with PBS containing 0.5% SDS.

Columns were plugged, and bound proteins were denatured and reduced by incubation with 200 µL PBS containing 0.5% SDS and 100 mM dithiothreitol (DTT; Invitrogen #D1532) for 20 min at room temperature (RT) with shaking at 200 rpm. Beads were subsequently washed 10 times with 500 µL 6 M urea buffer (6 M urea, 100 mM Tris‐Cl, pH 8.5) to remove SDS. Proteins were alkylated with 200 µL 6 M urea buffer containing 50 mM chloroacetamide (CAA) for 20 min at RT with shaking at 200 rpm.

Following reduction and alkylation, beads were washed 10 times with 500 µL 6 M urea buffer, four times with 500 µL PBS, and three times with 500 µL Milli‐Q water. Bound proteins were digested on‐bead in 100 µL 50 mM ammonium bicarbonate (ABC) containing 1 µg SOLu‐trypsin (Sigma‐Aldrich #EMS0004) overnight at 37 °C with shaking at 200 rpm.

Peptides released from the beads after trypsinization were collected by centrifugation (1000×*g*, 1 min, RT) and acidified to a final concentration of 1% trifluoroacetic acid (TFA; Thermo Scientific #85183) before clarification by centrifugation (21,000 × *g*, 5 min, RT). Stage‐tips were prepared in‐house using two plugs of 3 M Empore SDB‐XC extraction disks (Sigma‐Aldrich #66884‐U), activated with 50 µL acetonitrile (ACN) and equilibrated with 50 µL 0.1% TFA and 2% ACN (1800 × *g*, RT). Clarified samples were loaded onto the stage‐tips and centrifuged (1800 × *g*, RT). Stage‐tips were washed once with 100 µL 0.1% TFA and 2% ACN before peptides were eluted with 100 µL 80% ACN and 0.1% TFA (1800 × *g*, RT). Eluates were dried using a CentriVap Benchtop Vacuum Concentrator (Labconco) and reconstituted in 15 µL 0.1% TFA and 2% ACN for mass spectrometry analysis.

### 4.8 No‐Enrichment Identification of Cleavage Events (NICE) for Mass Spectrometry

RAW264.7 cells were treated with either 0.1% DMSO or 5 µM Z‐RK‐AOMK (Section 4.2), harvested, and lysed in lysis buffer (4% SDS, 50 mM HEPES, pH 7.5) supplemented with complete EDTA‐free protease inhibitor cocktail (Roche #04693159001). Lysates were sonicated at 30% amplitude for 3 s (Qsonica Sonicator Q500), followed by heating at 95 °C for 10 min. Samples were clarified by centrifugation (21,000 × *g*, 7 min, 4 °C), and protein concentrations were determined using a BCA assay (Section 4.3). A 100 µg of protein per sample was adjusted to 100 µL in lysis buffer.

Proteins were reduced with 20 mM DTT (10 min, 80 °C, 500 rpm) and alkylated with 50 mM iodoacetamide (IAA) (30 min, 37 °C, 500 rpm). Excess IAA was quenched with 50 mM DTT (20 min, 37 °C, 500 rpm). Sera‐Mag SpeedBeads (GE Healthcare #45152105050250 and #65152105050250) were prepared by combining in a 1:1 ratio and washing three times with Milli‐Q water, then resuspending to 50 μg/μL in Milli‐Q water according to the SP3 protocol [75]. Conditioned SP3 beads were added at a protein‐to‐bead ratio of 1:20, and protein was precipitated by addition of ethanol to a final concentration of 80%. Samples were incubated for 20 min at 25 °C with shaking at 1000 rpm, followed by three washes with 80% ethanol on a magnetic rack. SP3 beads were then resuspended in 90 μL of 200 mM HEPES (pH 7.5).

Proteins were dimethylated by the addition of 30 mM formaldehyde (Sigma) and 30 mM sodium cyanoborohydride (Sigma), incubated for 1 h at 37 °C and shaking at 1000 rpm. This labeling step was repeated once to ensure complete dimethylation. Reactions were quenched by addition of 25 μL 4 M Tris‐base (pH 6.8) then incubated for 1 h at 37 °C with shaking at 1000 rpm. Samples were subjected to a second SP3 cleanup by adding conditioned SP3 beads at an additional protein‐to‐bead ratio of 1:10 and proteins re‐precipitated with 80% ethanol (20 min, 25 °C, 1000 rpm). After that, SP3 beads were washed three times with 80% ethanol and split into two equal aliquots per sample. SP3 beads were then resuspended in 50 μL of 200 mM HEPES (pH 7.5) and digested overnight at 37 °C with shaking at 1000 rpm using SOLu‐trypsin (trypsin:protein ratio 3:100; Sigma‐Aldrich #EMS0004).

Peptides were recovered from the SP3 beads using a magnetic rack, acidified with Buffer A (2% ACN, 0.1% TFA), and desalted using stage‐tips prepared in‐house with seven plugs of 3 M Empore C18 extraction disks (Sigma‐Aldrich #66883‐U) and OLIGO R3 reverse‐phase resin (Thermo Scientific #113390) [76]. Stage‐tips were activated with Buffer B (80% ACN, 0.1% formic acid) (2x, 2,500 *× g*, RT) and equilibrated with Buffer A (2x, 2500*×g*, RT). Acidified peptide samples were loaded and centrifuged (2500 × *g*, RT). Stage‐tips were then washed twice with Buffer A, and peptides were eluted twice with Buffer B. Eluates were dried using a CentriVap Benchtop Vacuum Concentrator (Labconco) and reconstituted in 30 µL Buffer A for mass spectrometry analysis.

### 4.9 Liquid Chromatography and Tandem Mass Spectrometry

LC‐MS/MS analysis of affinity purified samples (Section 4.7) was carried out using a Orbitrap Exploris 480 mass spectrometer (Thermo Scientific) equipped with a nanoflow reversed‐phase‐HPLC (Ultimate 3000 RSLC, Dionex) fitted with an Acclaim Pepmap nano‐trap column (Dionex—C18, 100 Å, 75 µm × 2 cm) and an Acclaim Pepmap RSLC analytical column (Dionex—C18, 100 Å, 75 µm × 50 cm). Tryptic peptides were injected into the trap column at an isocratic flow of 5 μL/min of 2% *v/v* acetonitrile containing 0.05% *v/v* trifluoroacetic acid for 6 min before the trap column was switched in‐line with the analytical column. The eluents were 5% DMSO in 0.1% *v/v* formic acid (solvent A) and 5% DMSO in 100% *v/v* CH3CN and 0.1% *v/v* formic acid (solvent B). The flow gradient was (i) 0–6 min at 3% B, (ii) 6–35 min at 3%–35% B (ii) 35–45 min at 35%–45% B, (iii) 45–50 min at 45%–80% B, (iv) 50–55 min at 80%–80% B, (v) 55–55.1 min at 80%–3% B, and (vi) 55.1–65 min at 3%–3% B. For data‐dependent acquisition (DDA) experiments full MS resolutions were set to 120,000 at m/z 200 and scanning from 300–1600 m/z in the profile mode. Full MS automated gain control (AGC) target was 300% with an IT of 25 ms. MS/MS spectra were acquired using a 3 s cycle time. Ions with charge states of 2–6 and an intensity over 5e3 were isolated using the quadrupole with a width of 1.2 m/z. Selected ions were fragmented with higher‐energy collisional dissociation (HCD) using a normalized collision energy of 30%. Analysis of fragment ions was carried out in the Orbitrap in the positive mode at 15,000 resolutions over the “Normal” mass range. AGC was set to standard with a normalized value of 75%. Dynamic exclusion was activated for 20 s with a mass tolerance of 10 ppm.

LC MS/MS analysis of NICE samples (Section 4.8) was performed on an Orbitrap Astral (ThermoFisher Scientific). The LC system was a Vanquish Neo UHPLC (Thermo Scientific) and using the heated trap and elute setup. The trap column was an Acclaim Pepmap nano‐trap column (Dionex—C18, 100 Å, 75 µm × 2 cm). The analytical column was a 5.5 cm high‐throughput (HTP) µPAC Neo analytical column (Thermo Scientific). The eluents were water with 0.1% v/v FA (solvent A) and 80% CH3CN with 0.1% v/v FA acid (solvent B). The analytical column flow rate was 750 nL/min and the gradient was (i) 0–0.3 min, 3%–6% B; (ii) 0.3–16 min, 6%–23.5% B; (iii) 16–17.7 min, 23.5%–40% B; (iv) 17.7–‍18.7 min, 40%–50% B; and (v) 18.7–18.8 min, 50%–99% B. Column wash was then activated at 2 µL/min (combine flow and pressure control) for 1.2 min followed by trap column wash (combine flow and pressure control with 2 zebra wash cycles at 80% CH3CN) and fast equilibration of the analytical column (combine flow and pressure control). For data‐independent acquisition (DIA), full MS resolutions were set to 120,000 at m/z 200 and scanning from 380–980 m/z in the profile mode. Full MS AGC target was 500% with a maximum IT of 5 ms. DIA was carried out in the Astral analyser with isolation window of 2 m/z, normalized HCD collision energy of 27, normalized AGC target of 500% and maximum injection time of 3 ms. The cycle time was kept to 0.6 s.

### 4.10 Mass Spectrometry Data Analysis

Data‐dependent acquisition (DDA) mass spectra from affinity‐purified DCG‐04‐labeled samples were processed using FragPipe (version 23.1) [77] via the default LFQ_MBR workflow. Raw files were searched using MSFragger (version 4.4.1) [77] against the reviewed *Mus musculus* UniProt proteome (proteome ID: UP000000589; downloaded December 2025; 51,538 entries), supplemented with common contaminants and a reverse decoy database. Match between runs (MBR) was disabled during database searching. The resulting combined_protein.tsv output was imported into Perseus (version 2.1.5.0) [78] for downstream analysis. Proteins not assigned to *Mus musculus* were removed, and LFQ intensities were log_2_‐transformed. Samples were grouped according to the experimental conditions (*n* = 4), and proteins with ^3^ 3 valid values in at least one group were retained. Missing values were imputed in Perseus from a normal distribution for each sample (width = 0.3, and down shift = 1.8). Statistical significance was determined using unpaired t‐tests with a false discovery rate (FDR) of 0.05. The processed data were subsequently visualized as a volcano plot using R (version 4.5.3).

Data‐independent acquisition (DIA) mass spectra from NICE were processed using FragPipe (version 23.1) [77] via the DIA_SpecLib_Quant workflow. Raw files were searched using MSFragger (version 4.4.1) [77] against a reviewed *Mus musculus* UniProt‐proteome (proteome ID: UP000000589; downloaded December 2025; 51,538 entries), supplemented with common contaminants and a reverse decoy database. Cysteine carbamidomethylation (+57.0215 Da) was specified as a fixed modification. Variable modifications included lysine dimethylation (+28.0313 Da), N‐terminal dimethylation (+28.0313 Da), methionine oxidation (+15.9949 Da), N‐‍terminal acetylation (+42.0106 Da), and N‐‍‍terminal cyclization (−17.0265/‐18.0106 Da). Enzymatic cleavage specificity was defined according to the analysis type: “ENZYMATIC” for global proteomics, “SEMI_N_TERM” for N‐terminomics, and “SEMI” for C‐terminomics [42]. Trypsin (“trypsin_r”) was specified, allowing up to one missed cleavage. Peptide‐spectrum matches (PSMs) were rescored using MSBooster (version 1.3.17) [79] and Percolator (version 3.7.1) [79]. Peptide and protein‐level FDR were controlled at 1% using Philosopher (version 5.1.2) [80]. Empirical spectral libraries generated from the MSFragger outputs were used in DIA‐NN (version 1.8.2 beta 8) [81] for peptide identification and quantification using default settings.

The resulting DIA‐NN outputs were further processed in R (version 4.5.3). Common contaminants and reverse decoys were removed before log_2_‐transformation of intensity values. Proteins and peptides detected in at least three out of four biological replicates in at least one experimental group were retained for downstream analysis. For N‐terminomic and C‐terminomic datasets, peptide abundances were additionally normalized to protein abundance to account for variation in protein expression. This was performed by summing precursor intensities at the modified sequence level (N‐termini) or stripped peptide sequence level (C‐termini), followed by calculation of log_2_(peptide intensity) – log_2_ (protein abundance). Missing values were imputed using a hybrid approach reflecting underlying missingness mechanisms. Values classified as missing not at random (MNAR), defined as peptides or proteins with ≥3 missing values per experimental group, were imputed using left‐censored imputation (QRILC). Remaining missing values, assumed to be missing at random (MAR), were imputed using a random forest‐based method [82, 83]. Neo‐N‐termini were defined as peptides carrying N‐terminal dimethylation that did not correspond to the annotated protein N‐terminus. Only peptides containing fully dimethylated internal lysine residues were included to ensure confident assignment of N‐terminal labeling. Neo‐C‐termini were defined as non‐native protein C‐termini that did not correspond to expected tryptic cleavage sites. Differential abundance analysis was performed using the limma package in R [52, 84, 85]. Statistical significance was defined as a |log_2_ fold‐change| ≥ 0.585 (fold‐change of 1.5) and an adjusted *p* value (Benjamini–Hochberg FDR) <0.05 derived from limma statistics.

### 4.11 General Chemistry Procedures

All non‐aqueous reactions were performed under an atmosphere of nitrogen, unless otherwise specified. Commercially available reagents were used without further purification. Flash chromatography was performed with silica gel 60 (particle size 0.040–0.063 μm) on a CombiFlash Rf Purification System (Teledyne Isco) with mobile phase gradients as specified. NMR spectra were recorded on a Bruker Avance DRX 300 with the solvents indicated (^1^H NMR at 300 MHz). Chemical shifts are reported in ppm on the δ scale and referenced to the appropriate solvent peak. LCMS analysis was acquired on an Agilent LCMS system equipped with an Agilent G6120B Mass Detector, 1260 Infinity G1312B Binary pump, 1260 Infinity G1367E HiPALS autosampler, and 1260 Infinity G4212B Diode Array Detector. The LCMS conditions were as follows: column, Luna Omega (1.6 µm, C18, 50 × 2.1 mm); injection volume, 1 µL; gradient, 5%–100% B over 3.8 min (solvent A: water/0.1% formic acid; solvent B: ACN/ 0.1% formic acid); acquisition time, 4.1 min; flow rate, 1 mL/min; and detection, 254 and 214 nm. Unless otherwise noted, all compounds were found to be >95% pure by this method. The preparative LCMS purification was performed using a Waters preparative HPLC system equipped with Waters ZQ 3100 –Mass Detector, Waters 2545‐Pump, Waters SFO System Fluidics Organizer, Waters 2996 Diode Array Detector, and Waters 2767 Sample Manager. The preparative‐HPLC conditions were as follows: XBridge BEH C18 OBD Prep Column (130 Å, 5 µm, 19 mm × 100 mm); injection volume, 1 mL; gradient is variable over 20 min depending on each compound (solvent A, water/0.1% formic acid; solvent B, ACN/ 0.1% formic acid); flow rate, 20 mL/min; detection 100–600 nm. HRMS were acquired through the Bio21 Mass Spectrometry and Proteomics Facility using a Thermo Scientific Orbitrap Exactive mass spectrometer.

## Funding

This work was supported by the National Health and Medical Research Council (2011119).

## Conflicts of Interest

The authors declare no conflicts of interest.

## Supporting information

Supplementary Material

Supplementary Material

Supplementary Material

Supplementary Material

Supplementary Material

Supplementary Material

Supplementary Material

Supplementary Material

Supplementary Material

## Data Availability

The raw proteomics data from this study are openly available in PRIDE ProteomeXchange at https://www.ebi.ac.uk/pride/markdownpage/proteomexchange, Reference Numbers PXD081856 and PXD081779. All other data are provided within the manuscript.
