# Supplementary material for: Integration of Chemical Probes and Degradomics Reveals That Cathepsin B Functions Primarily as a Dipeptidyl Carboxypeptidase in Macrophages
Source: ChemMedChem. 2026 Oct 29;21(21):e70530. doi: 10.1002/cmdc.70530 (PMC13719178; doi:10.1002/cmdc.70530)
Supplement: Supplementary file 1 [file CMDC-21-e70530-s008.pdf]

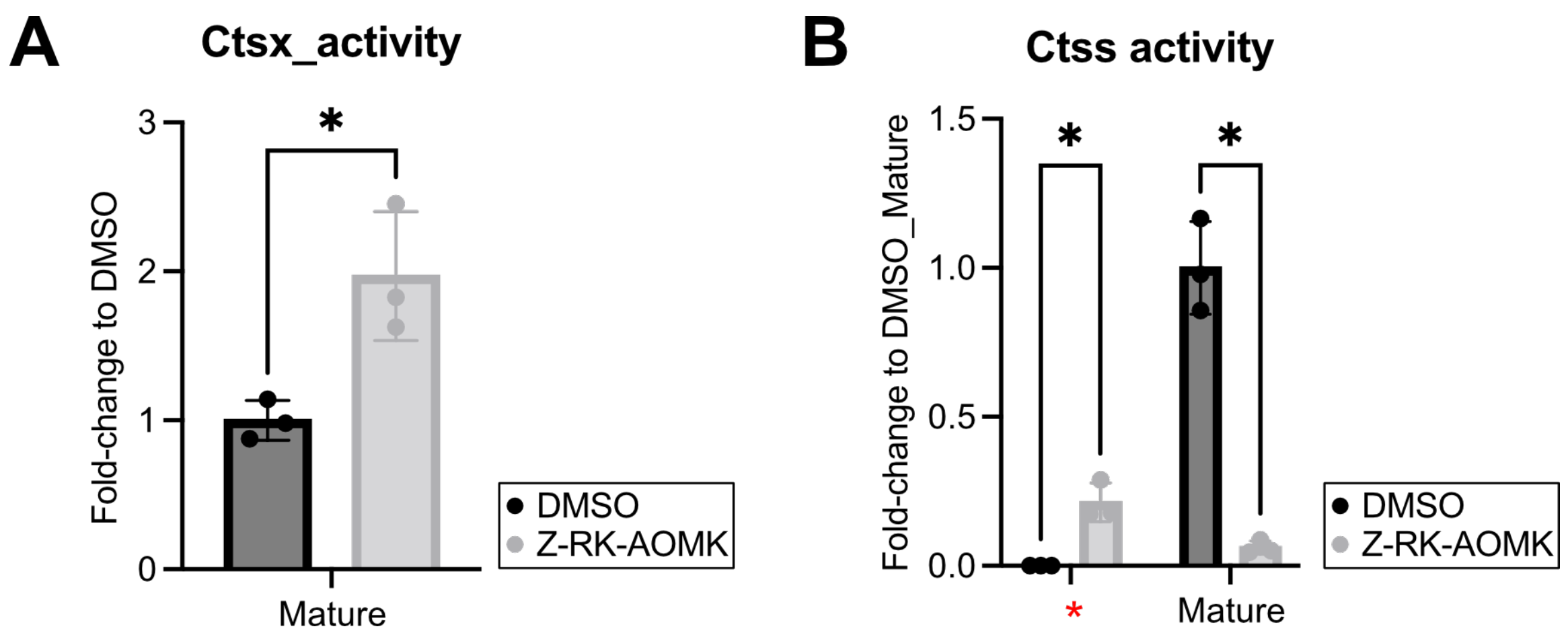

Figure S1. Activities of Ctsx and Ctss detected by sCy5-Nle-SY upon Z-RK-AOMK treatment.

(A) Quantification of Ctsx labelling by sCy5-Nle-SY shown in Figure 2F. Signal intensities were normalised to β-actin loading control and shown as fold-change relative to DMSO-treated cells (set to 1). Data represent mean ± SD from n = 3 biological replicates. Statistical significance was assessed using unpaired t-tests in Prism 10 (ns = not significant; *$P < 0.05$).

(B) Quantification of Ctss labelling by sCy5-Nle-SY shown in Figure 2F. Signal intensities were normalised to β-actin loading control and shown as fold-change relative to the mature form of DMSO-treated cells (set to 1). Data represent mean ± SD from n = 3 biological replicates. Statistical significance was assessed using unpaired t-tests in Prism 10 (ns = not significant; *$P < 0.05$).

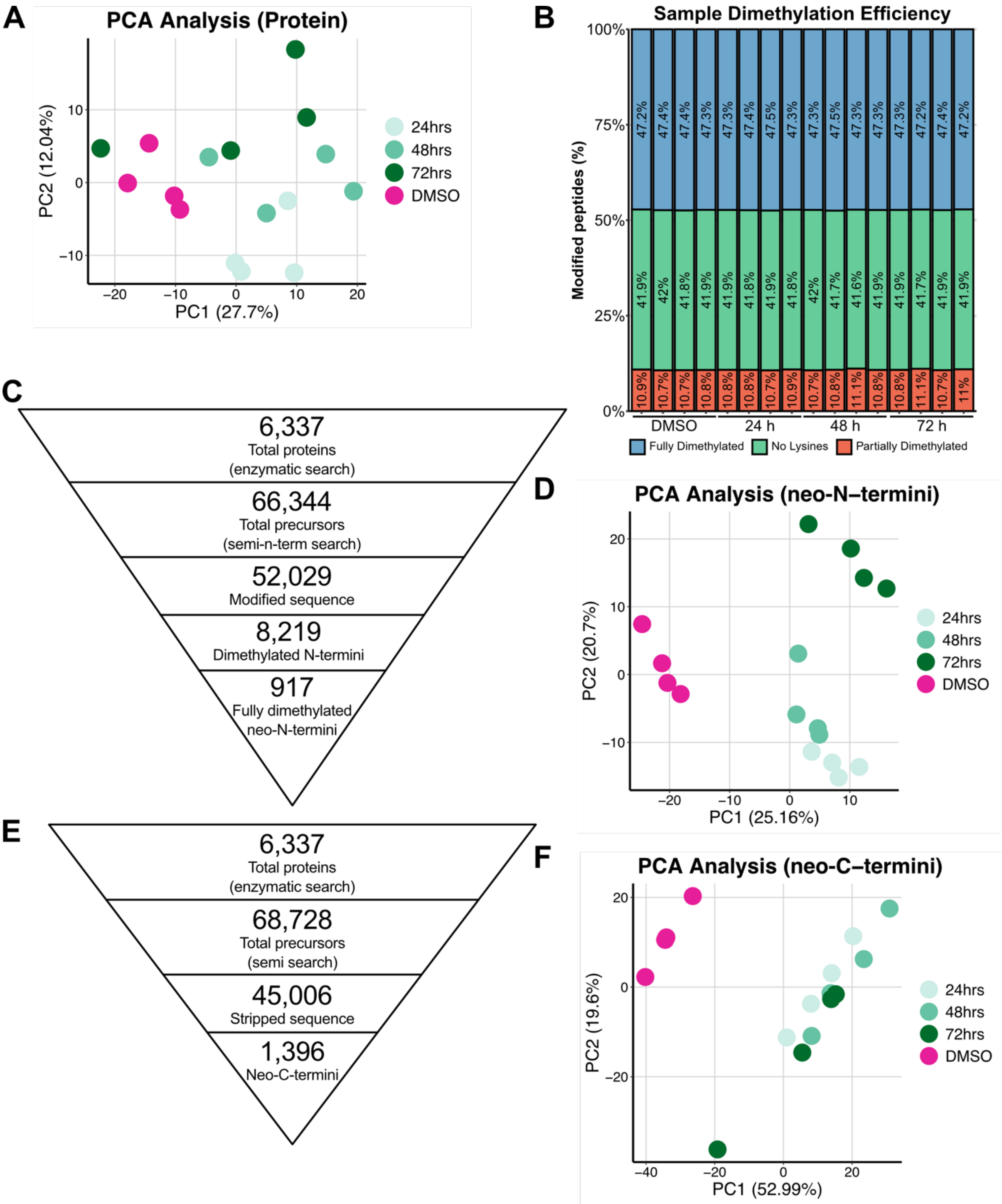

Figure S2. NICE data quality control and summary.

(A) Principal component analysis (PCA) of Log_2-transformed and imputed LFQ protein abundance values from proteomics analysis of samples shown in Figure 4A-C.

(B) Dimethylation efficiency of each sample, assessed by the proportion of detected peptides containing partial or complete lysine dimethylation as a proxy for labelling efficiency.

(C) Total protein and neo-N-termini identification from the NICE workflow. Proteins were searched using fully tryptic specificity (enzymatic), while raw files were additionally searched using semi-specific N-terminal specificity (semi-n-term) to enable identification of neo-N-terminal peptides. Peptide-spectrum matches were collapsed based on modified peptide sequence with intensities summed, followed by filtering for N-terminal dimethylation to identify endogenous N-termini. Identified N-termini were further filtered for complete dimethylation and exclusion of annotated protein N-termini to define neo-N-termini. Differential abundance analysis was subsequently performed on neo-N-terminal peptides, with resulting volcano plots shown in Figure 5A–C.

(D) Principal component analysis (PCA) of Log_2-transformed, protein abundance-normalised, and imputed LFQ neo-N-termini abundance values from N-terminomic analysis of samples shown in Figure 5A-C.

(E) Total protein and neo-C-termini identification from the NICE workflow. Proteins were searched using fully tryptic specificity (enzymatic), while raw files were additionally searched using semi-specific cleavage specificity (semi) to enable identification of neo-C-termini. Peptide-spectrum matches were collapsed based on stripped peptide sequence with intensities summed, followed by filtering for neo-C-termini by excluding peptides with C-terminal arginine cleavage and peptides correspond to annotated native protein C-termini. Differential abundance analysis was subsequently performed on neo-C-termini, with resulting volcano plots shown in Figure 6A–C.

(F) Principal component analysis (PCA) of Log_2-transformed, protein abundance-normalised, and imputed LFQ neo-C-termini abundance values from C-terminomic analysis of samples shown in Figure 6A-C.

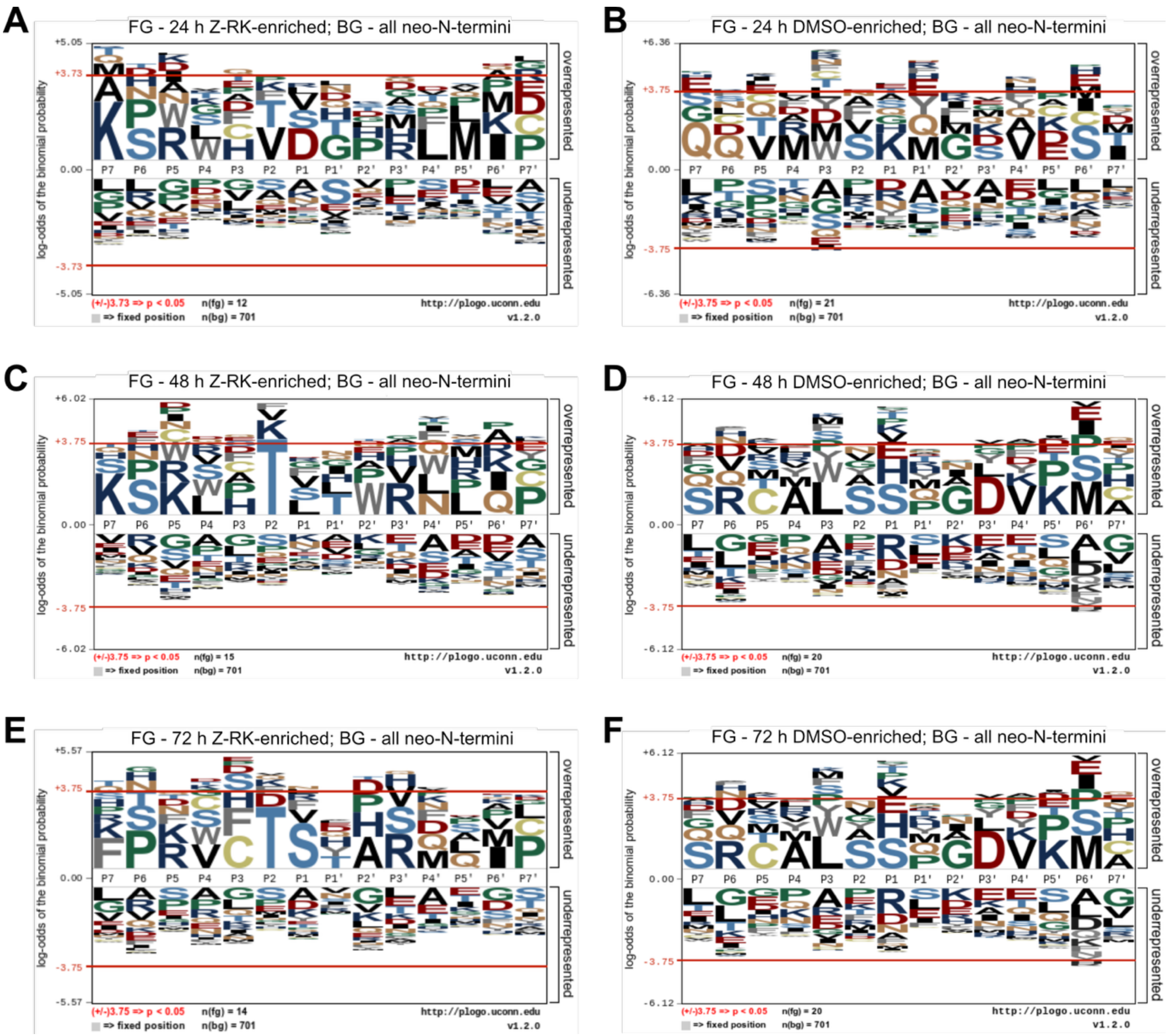

Figure S3. pLogo analysis of P7–P7′ flanking sequences of significantly differentially enriched neo-N-termini identified in Figure 5A–C. The P7–P7′ flanking sequences of all identified neo-N-termini were used as the background distribution for this analysis.

(A) Neo-N-termini enriched in 24 h Z-RK-AOMK-treated cells relative to DMSO-treated cells (Figure 5A).

(B) Neo-N-termini enriched in DMSO-treated cells relative to 24 h Z-RK-AOMK-treated cells (Figure 5A).

(C) Neo-N-termini enriched in 48 h Z-RK-AOMK-treated cells relative to DMSO-treated cells (Figure 5B). Threonine (T) at the P2 position was significantly enriched (33.33%, log-odds score = 3.975, $p = 0.03$).

(D) Neo-N-termini enriched in DMSO-treated cells relative to 48 h Z-RK-AOMK-treated

cells (Figure 5B).

(E) Neo-N-termini enriched in 72 h Z-RK-AOMK-treated cells relative to DMSO-treated cells (Figure 5C).

(F) Neo-N-termini enriched in DMSO-treated cells relative to 72 h Z-RK-AOMK-treated cells (Figure 5C).

(A) ^{1}H-NMR

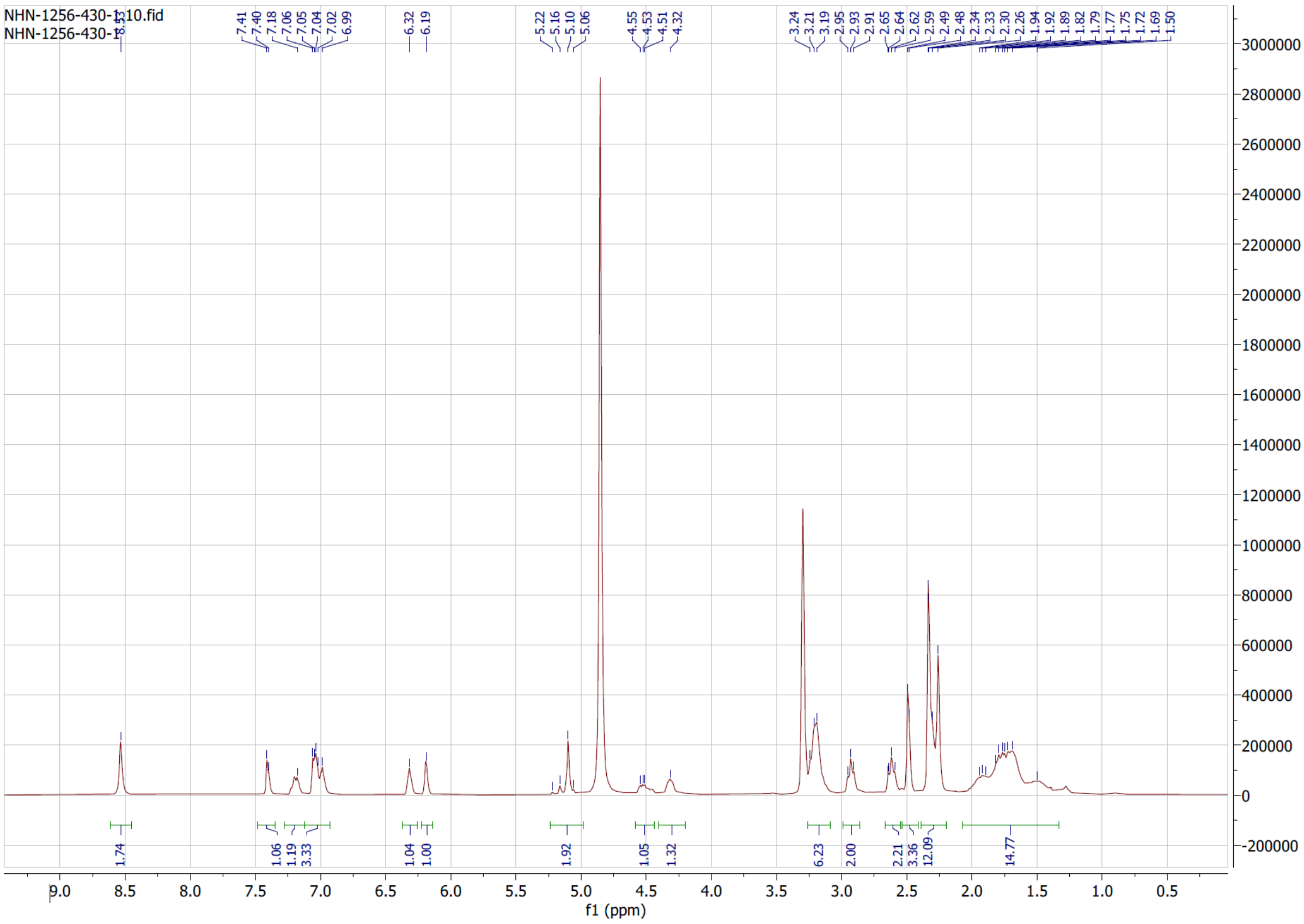

(B) ^{13}C-NMR

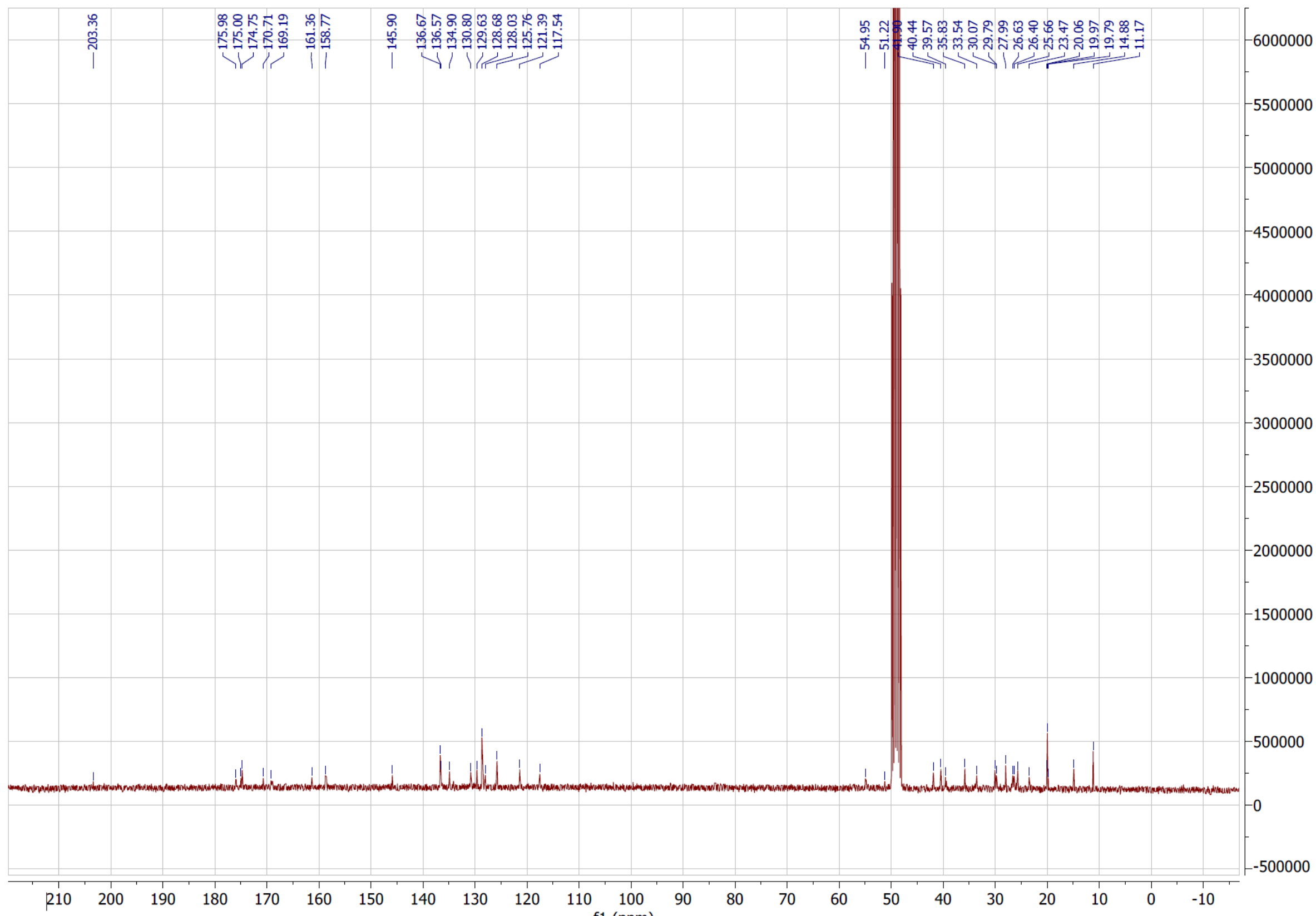

(C) LC trace

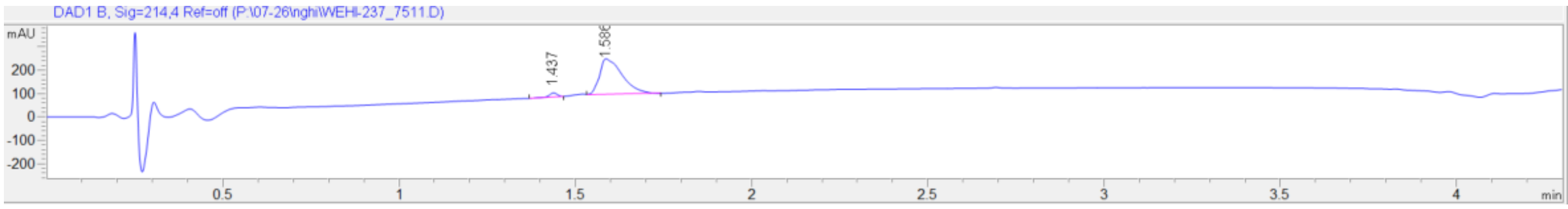

(D) HRMS

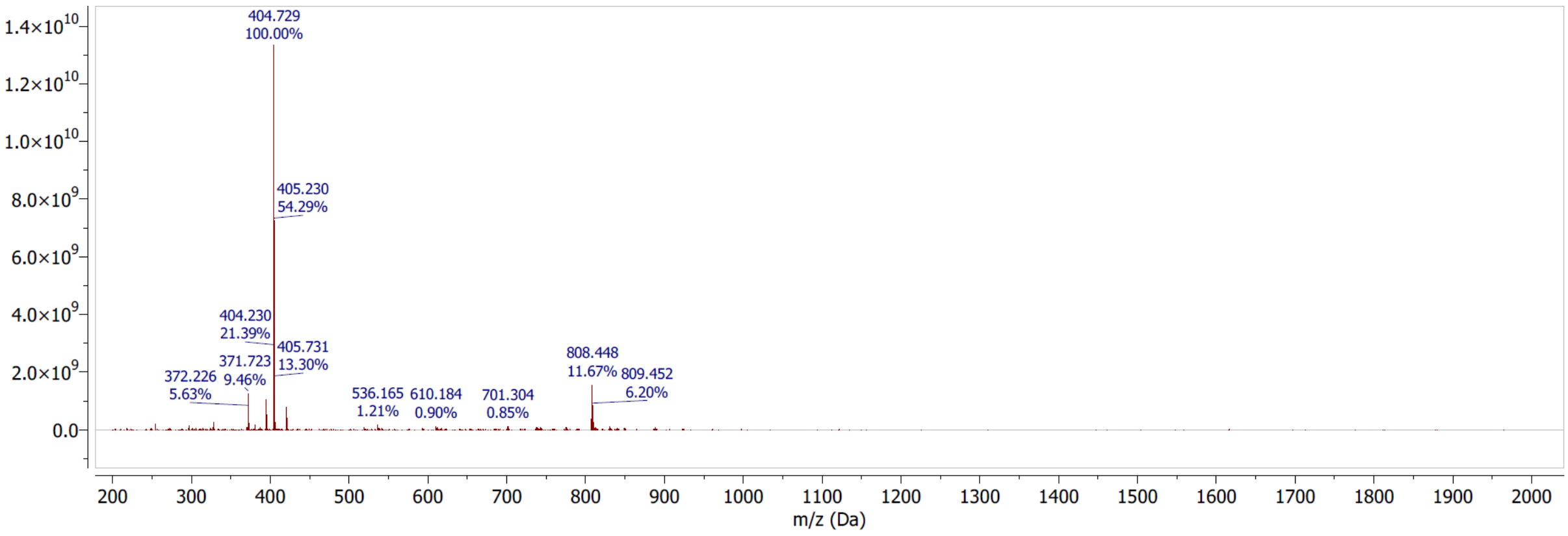

Figure S4. Characterisation of WEHI-237 (BODIPY-RK-AOMK).

(A) ^{1}H-NMR spectrum.

(B) ^{13}C-NMR spectrum.

(C) LC trace.

(D) HRMS.

Supplementary Method. Synthesis of Z-RK-AOMK and BODIPY-RK-AOMK.

(*S*)-3-((((9*H*-Fluoren-9-yl)methoxy)carbonyl)amino)-7-((*tert*-butoxycarbonyl)amino)-2-oxoheptyl 2,6-dimethylbenzoate 3. A mixture of KF (390 mg, 6.7 mmol) and 2,6-dimethylbenzoic acid (500 mg, 3.3 mmol) in anhydrous DMF (2 mL) was sonicated for 10 min. Compound **1** (910 mg, 1.7 mmol) dissolved in DMF (1 mL) was added dropwise to stirred of the reaction mixture. After 30 min, the solution was diluted with ethyl acetate (50 mL) washed twice with water and brine and dried over $MgSO_4$ and concentrated. The crude oil was purified by flash chromatography eluting with 0-20% EtOAc/heptane to give compound **3** as an off-white solid (0.96 g, 47% yield). LC-MS (ESI) t_R 2.94 min, *m*/*z* 515.6 [M-Boc]. ^{1}H NMR (300

MHz, $CDCl_3$) δ 7.77 (d, *J* = 7.4 Hz, 2H), 7.61 (d, *J* = 7.4 Hz, 2H), 7.40 (tdd, *J* = 7.5, 1.3, 0.6 Hz, 2H), 7.32 (td, *J* = 7.4, 1.3 Hz, 2H), 7.21 (dd, *J* = 8.2, 7.0 Hz, 1H), 7.08 – 7.02 (m, 2H), 5.53 (d, *J* = 7.6 Hz, 1H), 5.10 – 4.88 (m, 2H), 4.64 (s, 1H), 4.55 – 4.37 (m, 3H), 4.22 (t, *J* = 6.6 Hz, 1H), 3.19 – 3.04 (m, 2H), 2.40 (s, 6H), 2.07 – 1.88 (m, 1H), 1.81 – 1.61 (m, 2H), 1.61 – 1.34 (m, 13H). ^{13}C NMR (75 MHz, $CDCl_3$) δ 202.3, 169.2, 156.4, 156.3, 143.8, 141.5, 135.8, 132.5, 129.9, 127.9, 127.8, 127.2, 125.2, 125.2, 120.1, 79.4, 67.1, 66.7, 57.5, 47.4, 39.9, 30.9, 29.8, 28.5, 22.1, 20.0.

(*S*)-3-((*S*)-2-((((9*H*-Fluoren-9-yl)methoxy)carbonyl)amino)-5-(3-((2,2,4,6,7-pentamethyl-2,3-dihydrobenzofuran-5-yl)sulfonyl)guanidino)pentanamido)-7-((*tert*-butoxycarbonyl)amino)-2-oxoheptyl 2,6-dimethylbenzoate 4. DBU (0.067 mL, 0.45 mmol) was added to a stirred solution of compound **3** (230 mg, 0.37 mmol) in DCM (3 mL). The reaction solution was stirred until the Fmoc was fully deprotected ~10 min (monitored by LCMS). To this solution, Fmoc-Arg(Pbf)-OH (200 mg, 0.31 mmol), HATU (140 mg, 0.37 mmol), DIPEA (0.161 mL, 0.925 mmol) in DCM (5 mL) were added. The reaction solution was then stirred at r.t for 30 min. The reaction mixture was washed with water, brine, dried over $MgSO_4$ and concentrated. The crude product was purified by flash chromatography eluting with 10-100% EtOAc/heptane to give compound **4** as an off-white solid (159 mg, 50% yield). The product was further purified by preparative HPLC (30-80% CH_3CN/H_2O/0.1% formic acid) and lyophilized. LC-MS (ESI) t_R 3.10 min, *m*/*z* 1024.6 $[M+H]^+$. ^1H NMR (300 MHz, $CDCl_3$) δ 7.80 (s, 1H), 7.72 (d, *J* = 7.5 Hz, 2H), 7.56 (t, *J* = 6.9 Hz, 2H), 7.35 (t, *J* = 7.5 Hz, 2H), 7.28 – 7.12 (m, 4H), 7.00 (d, *J* = 7.6 Hz, 2H), 6.52 (s, 2H), 6.16 (s, 1H), 5.05 (s, 2H), 4.83 (s, 1H), 4.54 (s, 1H), 4.47 – 4.25 (m, 3H), 4.22 – 4.08 (m, 1H), 3.29 (s, 2H), 3.01 (s, 1H), 2.89 (s, 2H), 2.58 (s, 3H), 2.50 (s, 3H), 2.33 (s, 6H), 2.05 (s, 3H), 1.90 (s, 2H), 1.84 – 1.69 (m, 2H), 1.61 (s, 2H), 1.52 – 1.25 (m, 20H). ^{13}C NMR (75 MHz, $CDCl_3$) δ 202.4, 172.9, 169.1, 159.2, 156.6, 156.3, 143.9, 143.7, 141.3, 141.2, 138.8, 135.7, 132.5, 129.7, 127.7, 127.7, 127.1, 125.2, 125.2, 124.9, 120.0, 117.8, 86.6, 79.2, 77.3, 67.2, 66.9, 56.5, 54.0, 47.1, 43.1, 40.0, 30.0, 29.6, 28.6, 28.4,

25.2, 22.7, 19.9, 19.4, 18.0, 12.5.

Preparation of 7-(3-((3-Carboxypropyl)amino)-3-oxopropyl)-5,5-difluoro-1,3-dimethyl-5*H*-dipyrrolo[1,2-c:2',1'-f][1,3,2]diazaborinin-4-ium-5-uide 5. DIPEA (0.537 mL, 3.08 mmol) was added to a stirred solution of 3-BODIPY-propanoic acid (300 mg, 1 mmol), HATU (430 mg, 1.1 mmol) and *tert*-butyl 4-aminobutanoate.HCl (220 mg, 1.1 mmol) in DCM (10 mL). The reaction solution was then stirred at r.t. for 30 min. The volatile solvent was removed *in vacuo* and purified by flash chromatography, eluted with 0-60% EtOAc/heptane to give the *tert*-butyl ester intermediate as a red solid (310 mg, 70% yield). LCMS t_R 2.31 min, *m/z* 432.8 $[M-H]^-$. The *tert*-butyl ester intermediate (200 mg, 0.21 mmol) was dissolved in DCM (1 mL) and TFA (400 mL) was then added. The reaction solution was then stirred for 30 min at r.t. The volatile solvent was removed *in vacuo* and purified by flash chromatography eluting with 0-5% MeOH/DCM to give compound **5** as a red solid (110 mg, 63% yield). The product was further purified by preparative HPLC (5-30% CH_3CN/H_2O/0.1% formic acid) and lyophilized. LCMS t_R 1.83 min, *m/z* 376.6 $[M-H]^-$. ^1H NMR (300 MHz, DMSO) δ 12.05 (s, 1H), 7.94 (t, *J* = 5.7 Hz, 1H), 7.69 (s, 1H), 7.08 (d, *J* = 4.0 Hz, 1H), 6.34 (d, *J* = 4.0 Hz, 1H), 6.30 (s, 1H), 3.07 (dt, *J* = 8.0, 4.9 Hz, 4H), 2.48 – 2.42 (m, 5H), 2.31 – 2.18 (m, 5H), 1.62 (p, *J* = 7.2 Hz, 2H). ^{13}C NMR (75 MHz, DMSO) δ 174.2, 170.7, 159.1, 157.9, 144.1, 134.4, 133.0, 128.9, 125.3, 120.2, 116.6, 37.9, 33.8, 31.0, 24.6, 24.0, 14.5, 11.0.

7-((10*S*,13*S*)-10-(2-((2,6-Dimethylbenzoyl)oxy)acetyl)-2,2-dimethyl-4,12,15,20-tetraoxo-13-(3-(3-((2,2,4,6,7-pentamethyl-2,3-dihydrobenzofuran-5-yl)sulfonyl)guanidino)propyl)-3-oxa-5,11,14,19-tetraazadocosan-22-yl)-5,5-difluoro-1,3-dimethyl-5*H*-dipyrrolo[1,2-c:2',1'-f][1,3,2]diazaborinin-4-ium-5-uide 6. DBU (0.0302 mL, 0.220 mmol) was added to a stirred solution of **4** (150 mg, 0.15 mmol) in DCM (2 mL). The reaction mixture was stirred until the Fmoc was fully deprotected ~10 min (monitored by LCMS). Compound **5** (50.0 mg, 0.133

mmol), HATU (55.4 mg, 0.146 mmol) and DIPEA (0.0693 mL, 0.398 mmol) in DCM (3 mL) were then added to this solution. The reaction mixture was stirred at r.t for 40 min. The volatile solvent was removed *in vacuo* and purified by flash chromatography eluting with 0-10% MeOH/DCM to give compound **6** as a red solid (68 mg, 44% yield). The product was further purified by preparative HPLC (30-80% CH_3CN/H_2O/0.1% formic acid) and lyophilized. LCMS t_R 2.90 min, *m/z* 1161.6 $[M+H]^+$. ^1H NMR (300 MHz, $CDCl_3$) δ 7.95 (d, *J* = 9.0 Hz, 1H), 7.22 – 7.05 (m, 3H), 7.00 (d, *J* = 7.6 Hz, 2H), 6.87 (d, *J* = 4.0 Hz, 1H), 6.46 (d, *J* = 22.9 Hz, 3H), 6.24 (d, *J* = 4.1 Hz, 1H), 6.09 (s, 1H), 5.17 – 5.00 (m, 2H), 4.97 (d, *J* = 7.1 Hz, 1H), 4.51 (s, 2H), 3.40 – 2.96 (m, 8H), 2.92 (s, 2H), 2.68 – 2.45 (m, 12H), 2.34 (s, 6H), 2.22 (d, *J* = 2.6 Hz, 3H), 2.14 (s, 2H), 2.06 (s, 3H), 1.96 – 1.52 (m, 9H), 1.52 – 1.30 (m, 18H). ^{13}C NMR (75 MHz, $CDCl_3$) δ 202.4, 202.3, 173.5, 172.8, 169.2, 169.2, 160.6, 159.1, 157.1, 156.4, 144.3, 138.7, 135.8, 135.3, 133.4, 132.8, 129.7, 128.4, 127.7, 124.9, 124.1, 120.7, 117.8, 117.0, 86.7, 79.2, 77.4, 67.0, 56.7, 56.5, 53.0, 43.3, 40.3, 38.5, 35.3, 32.8, 29.6, 29.4, 28.7, 28.6, 25.7, 24.8, 22.8, 20.0, 19.5, 18.1, 15.1, 12.6, 11.4.

7-((5*S*,8*S*)-5-(4-Aminobutyl)-1-(2,6-dimethylphenyl)-8-(3-guanidinopropyl)-1,4,7,10,15-pentaoxo-2-oxa-6,9,14-triazaheptadecan-17-yl)-5,5-difluoro-1,3-dimethyl-5*H*-dipyrrolo[1,2-c:2',1'-f][1,3,2]diazaborinin-4-ium-5-uide formate salt WEHI-237. TFA (0.049 mL, 0.65 mmol) and TIPS (0.026 mL, 0.13 mmol) were added to a stirred solution of **6** (50 mg, 0.043 mmol) in DCM (1 mL). The reaction solution was stirred at r.t. for 1 h (monitored by LCMS) and the volatile solvent was removed *in vacuo*. The crude material was purified by preparative HPLC (5-30% CH_3CN/H_2O/0.1% formic acid) and lyophilized to give **WEHI-237** as a red solid (6.3 mg, 18% yield). LCMS t_R 1.59 min, *m/z* 808.8 $[M+H]^+$. ^1H NMR (300 MHz, MeOD) δ 8.53 (s, 2H), 7.40 (d, *J* = 4.4 Hz, 1H), 7.18 (s, 1H), 7.12 – 6.93 (m, 3H), 6.32 (s, 1H), 6.19 (s, 1H), 5.23 – 4.99 (m, 2H), 4.59 – 4.44 (m, 1H), 4.32 (s, 1H), 3.27 – 3.09 (m, 6H), 2.93 (t, *J* = 6.9 Hz, 2H), 2.62 (t, *J* = 7.0 Hz, 2H), 2.49 (d, *J* = 3.6 Hz, 3H), 2.39 – 2.20 (m, 11H), 2.07 – 1.33 (m, 15H). ^{13}C NMR (75 MHz, MeOD) δ 203.4, 176.0, 175.0, 174.7, 170.7, 169.2, 161.4,

158.8, 145.9, 136.7, 136.6, 134.9, 130.8, 129.6, 128.7, 128.0, 125.8, 121.4, 117.5, 55.0, 51.2, 41.9, 40.4, 39.6, 35.8, 33.5, 30.1, 29.8, 28.0, 26.6, 26.4, 25.7, 23.5, 20.1, 20.0, 19.8, 14.9, 11.2. HRMS *m*/*z*: [M+H] Calcd for $C_{40}H_{56}BF_2N_9O_6$ 808.4487; Found 808.4483.
